# Exploring the Link Between Periodontitis and Alzheimer’s Disease—Could a Nanoparticulate Vaccine Break It?

**DOI:** 10.3390/pharmaceutics17020141

**Published:** 2025-01-21

**Authors:** André Ferreira da Silva, Alexandra Gomes, Lídia M. D. Gonçalves, Adelaide Fernandes, António J. Almeida

**Affiliations:** Research Institute for Medicines (iMed.ULisboa), Faculty of Pharmacy, Universidade de Lisboa, Av. Professor Gama Pinto, 1649-003 Lisbon, Portugal; andreferreirasilva@ff.ulisboa.pt (A.F.d.S.); afgomes@ff.ulisboa.pt (A.G.); lgoncalves@ff.ulisboa.pt (L.M.D.G.); amaf@ff.ulisboa.pt (A.F.)

**Keywords:** Alzheimer’s disease, aetiology, periodontitis, *Porphyromonas gingivalis*, virulence factors

## Abstract

Alzheimer’s disease (AD) is the most prevalent neurodegenerative disorder, as approximately 55 million people worldwide are affected, with a significant tendency to increase. It reveals three main pathological features: amyloid plaques, neurofibrillary tangles, and neuroinflammation, responsible for the neurodegenerative changes that slowly lead to deterioration of personality and cognitive control. Over a century after the first case report, effective treatments remain elusive, likely due to an incomplete understanding of the precise mechanisms driving its pathogenesis. Recent studies provide growing evidence of an infectious aetiology for AD, a hypothesis reinforced by findings that amyloid beta functions as an antimicrobial peptide. Among the microorganisms already associated with AD, *Porphyromonas gingivalis* (*Pg*), the keystone pathogen of periodontitis (PeD), has received particular attention as a possible aetiological agent for AD development. Herein, we review the epidemiological and genetic evidence linking PeD and *Pg* to AD, highlighting the identification of periodontal bacteria in post mortem analysis of AD patients’ brains and identifying putative mechanistic links relevant to the biological plausibility of the association. With the focus on AD research shifting from cure to prevention, the proposed mechanisms linking PeD to AD open the door for unravelling new prophylactic approaches able to reduce the global burden of AD. As hypothesised in this review, these could include a bionanotechnological approach involving the development of an oral nanoparticulate vaccine based on *Pg*-specific antigens. Such a vaccine could prevent *Pg* antigens from progressing to the brain and triggering AD pathology, representing a promising step toward innovative and effective AD prevention.

## 1. Introduction

More than a century after the German psychiatrist Alöis Alzheimer first described the clinical and pathological features of “a peculiar severe disease process of the cerebral cortex”—later known as Alzheimer’s disease (AD) [1]—the world has seen remarkable health advancements that have significantly increased life expectancy [2]. Alongside the bright side of extended human longevity, however, emerges a concerning demographic and public health challenge as more people reach older ages [2], thereby facing a growing incidence and prevalence of age-related diseases such as AD—the most common cause of dementia (60–80% of all cases) [3]. AD is generally associated with ageing [4], rising persistently from around age 65 onwards [5]. Indeed, from an estimated 55 million people worldwide living with dementia in 2019, that number is projected to climb 153% to 139 million by 2050 [6], also representing a huge economic burden on the societal level, with an annual global cost of around a trillion dollars [7].

**Figure 1:**
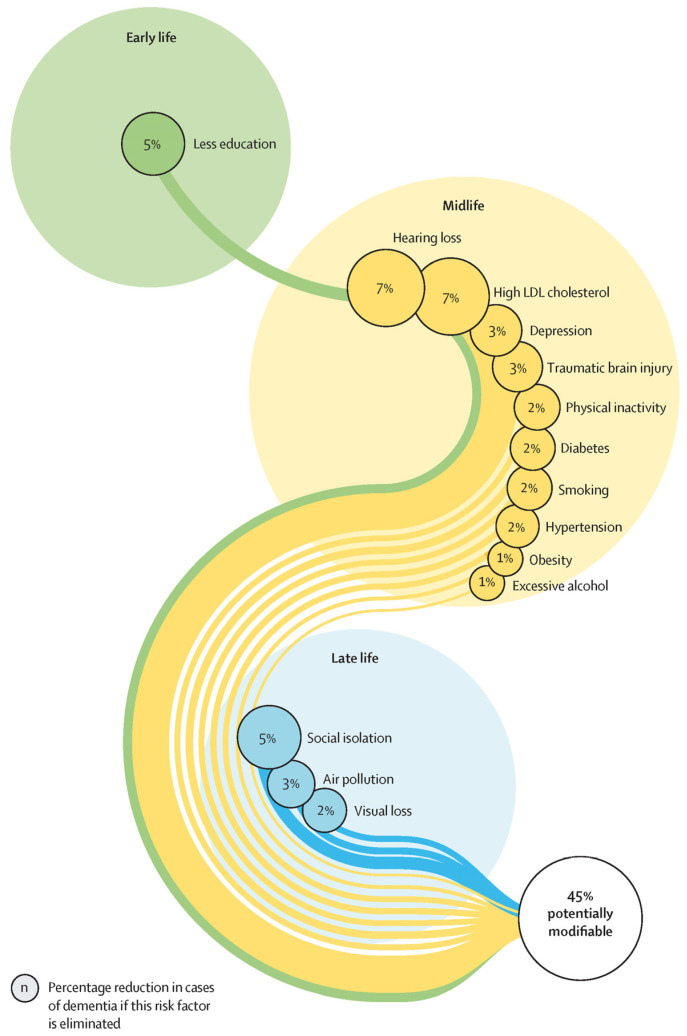
Life-course model of the contribution of modifiable risk factors to dementia from the *Lancet* Commission on Dementia 2024. Reprinted with permission from Ref. [10]. 2024, Elsevier.

Despite significant research efforts, there is still no effective disease-modifying or curative treatment for dementia. As the prospect of a “silver bullet” fades, the key to fighting dementia may lie in measures that either prevent it from developing in the first place or halt it before it causes irreversible damage [7]. Accumulating evidence suggests that dementia risk can be reduced through the management of potentially modifiable risk factors, indicating that prevention or delay is achievable via public health strategies and evidence-based interventions aimed at such factors [8,9,10]. In 2020, a *Lancet* report proposed a life-course model featuring 12 potentially modifiable risk factors for dementia, consolidating emerging evidence to outline specific targeted interventions for prevention [9]. More recently, The *Lancet* Commission on Dementia 2024 introduced an updated life-course model with 14 modifiable risk factors (Figure 1), estimating that these factors collectively account for roughly 45% of dementia cases, which could theoretically be prevented or delayed [10].

With the current focus on modifiable risk factors, an opportunity arises not only to refine interventions addressing established factors but also to consider new evidence on additional, potentially overlooked risk factors. Recent epidemiological, genetic, and preclinical studies have underscored a possible association between chronic periodontitis (PeD) and AD, suggesting that infection by the keystone pathogen *Porphyromonas gingivalis* (*Pg*) or other postulated periodontal pathogens may trigger the onset and development of AD [11,12,13,14]. Furthermore, building on findings that establish amyloid beta (Aβ)—the most studied hallmark protein in AD pathology—as a protective antimicrobial peptide (AMP) produced in the brain as part of an innate immune response to microbial infection [15,16], it is plausible to consider that infections caused by periodontal bacteria may significantly contribute to AD pathogenesis. Considering the growing body of evidence and support for this hypothesis, here we present a comprehensive review that examines PeD as a potential risk factor for AD, aiming to: (1) outline the most prominent hypotheses for AD causation, focusing on the recent antimicrobial protection hypothesis; (2) summarise the evidence linking PeD and *Pg* to AD; (3) elucidate the pathological mechanisms underpinning this association; and (4) introduce a novel nanotechnological approach to AD prevention.

## 2. Alzheimer’s Disease and Pathogenesis Hypotheses

AD is an age-related neurodegenerative disease characterised by progressive impairment of cognitive and behavioural functions, including orientation, memory, attention, language, visuospatial ability, judgement, problem-solving, and personality [17]. It progresses on a spectrum with three stages—an early, asymptomatic preclinical stage; a middle, pre-dementia stage of mild cognitive impairment (MCI); and a final stage characterised by symptoms of dementia [18]. AD cases can be further classified into two forms: familial (FAD) and sporadic (SAD). The former is related to uncommon hereditary cases of AD (~5%), where patients have autosomal-dominant mutations in selected genes (e.g., APP, PSEN1, and PSEN2), resulting in increased production of Aβ [19]. Even though both forms possess similar neuropathological hallmarks, FAD patients usually develop Alzheimer’s dementia earlier in life (<65) than patients with SAD (>65). Commonly referred to as late-onset AD, SAD comprises the majority (~95%) of all AD cases, progressing slowly with a long asymptomatic phase spanning more than one decade before the clinical onset of AD [20].

**Figure 2:**
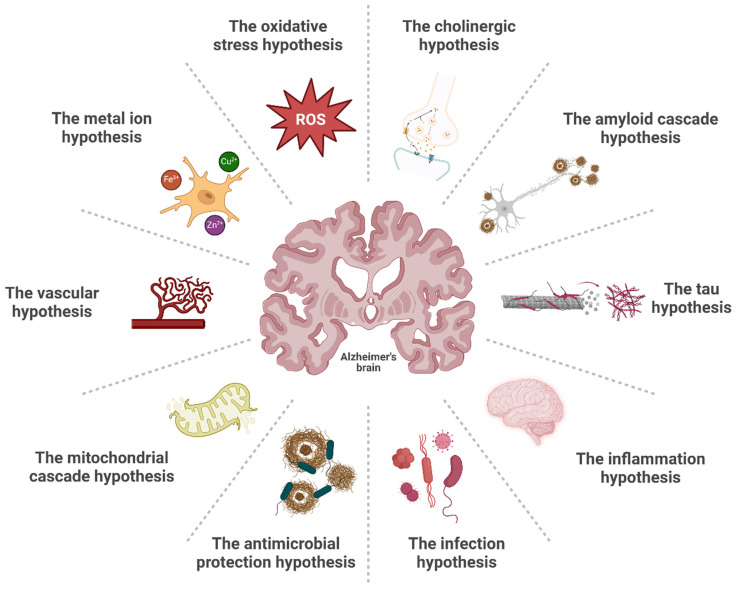
Overview of the major hypotheses of Alzheimer’s disease.

AD is believed to be a multifactorial disease resulting from a complex interplay between modifiable and unmodifiable risk factors. Among the latter, older age, family history of AD, and various gene polymorphisms appear to be the greatest risk factors for late-onset AD [21]. Starting decades prior to the onset of the clinical symptoms [22], neuroinflammation and the accumulation of extracellular amyloid plaques and intraneuronal neurofibrillary tangles (NFTs) containing hyperphosphorylated tau protein (p-tau) are the central neuropathological hallmarks of AD, leading to synaptic dysfunction and, ultimately, to neurodegeneration with consequential brain atrophy and cognitive decline [21]. Although no definitive explanation for the pathogenesis of the sporadic cases of the disease has been found, over the past decades, different hypotheses have been proposed to explain the driving mechanisms of AD (Figure 2).

### 2.1. The Cholinergic Hypothesis

In 1976, Davies and Maloney’s studies revealed a selective loss of central cholinergic neurones in AD, giving rise to the first of such hypotheses, the cholinergic hypothesis, which framed the disease as a failure of the cholinergic system [23]. Cholinergic neurotransmission, mediated by acetylcholine (ACh), plays a critical role in cognitive processes such as memory, learning, and attention. Consequently, its depletion, along with the degeneration of cholinergic neurones, is strongly associated with the cognitive dysfunction observed in AD patients. Based on this notion, acetylcholinesterase inhibitors (AChEIs) have been used in the past three decades to treat AD, owing to their ability to block the function of acetylcholinesterase (AChE), an enzyme that hydrolyses ACh at the synaptic cleft into acetate and choline. By inhibiting AChE, higher levels of ACh remain in the synaptic cleft, increasing the availability of ACh for synaptic transmission [24]. Capable of partially ameliorating cognitive symptoms, second-generation AChEIs such as donepezil, rivastigmine, and galantamine have been useful in improving the quality of life of patients, providing symptomatic relief in mild-to-moderate AD. However, these drugs are powerless to sustain their positive effects over time and to halt the underlying disease progression [25].

### 2.2. The Amyloid Cascade Hypothesis

The amyloid cascade hypothesis, the most studied and prominent one, portrays the mismetabolism of amyloid β precursor protein (APP), a transmembrane protein primarily found in synapses, and subsequent Aβ deposition in the brain as the primary events in the pathological cascade of AD, to which it succeeds the development of NFTs, depletion of transmitters, neuronal loss, and dementia [26,27]. The formation of amyloid plaques is associated with the amyloidogenic processing of APP. Under abnormal circumstances, the first proteolytic cleavage of APP is processed by β-secretase (BACE1) instead of the healthy, non-amyloidogenic proteolysis by α-secretase, leading to excess accumulation of Aβ peptide monomers (Aβ_42_ in particular) in the brain once a second proteolytic cleavage with γ-secretase occurs. This increased accumulation, coupled with a decline in Aβ clearance, progressively results in Aβ aggregation into oligomers, protofibrils, and fibrils, which finally deposit into the classical aggregates as extracellular senile plaques, capable of directly or indirectly damaging neurones and interfering with synaptic communication [17,28].

Moreover, these Aβ deposits induce a microglia-mediated local inflammatory reaction that activates astrocytes, triggering oxidative imbalance and the production of proinflammatory mediators. Under chronic inflammatory response, these mediators induce synaptic and neuronal damage, impaired blood–brain barrier (BBB), and tau hyperphosphorylation. Aβ itself may also mediate the activation of kinases involved in the regulation of tau phosphorylation, such as glycogen synthase kinase 3 beta (GSK3β), thus promoting tau hyperphosphorylation and the formation of neurotoxic NFTs. These events are likely to occur in a positive feedback loop, enhancing Aβ hazardous effects and culminating in neurodegeneration and disease progression [28,29]. Furthermore, soluble oligomeric forms of Aβ may also spread to multiple brain regions and trigger harmful neuropathological changes [30,31]. Despite all the evidence, most candidate Aβ-directed therapies have failed to achieve clinical relevance, with recent exceptions like aducanumab and lecanemab showing promising but limited success [32].

### 2.3. The Tau Hypothesis

Considering the numerous failures of Aβ-directed therapies and the stronger correlation of tau pathology with cognitive impairment and AD severity [33], a competing tau hypothesis has emerged, proposing tau pathology rather than Aβ as the primary trigger of the neuropathological events found in AD. Primarily found in the neuronal body, tau tangles are mainly composed of abnormally phosphorylated tau, a microtubule-stabilising protein whose phosphorylation state influences its interaction with tubulin. Under abnormal hyperphosphorylation, tau loses its ability to bind and stabilise tubulin polymers, detaches from the microtubule structure, and concentrates in the cytosol, consequently leading to defective microtubule functioning and progressive assembly of hyperphosphorylated tau into neurotoxic NFTs, with consequent cytoskeletal breakdown and neuronal death [17,28]. Despite promising preclinical results, tau-directed therapies have generally faced clinical difficulties similar to those observed in Aβ-targeting trials, requiring further demonstration of their clinical efficacy [25,29].

Given the limitations of Aβ and tau cascades in explaining the full spectrum of clinical outcomes and pathological events associated with the disease continuum, alternative hypotheses have emerged (e.g., the mitochondrial cascade hypothesis [34], the vascular hypothesis [35], the metal ion hypothesis [36], the oxidative stress hypothesis [37], and others [38,39]). These theories propose upstream mechanisms that may drive both cascades and the broader disease process, offering varied perspectives on potential key drivers of pathology.

### 2.4. The Inflammation Hypothesis

Compelling evidence from genetic and transcriptomic studies linking several microglial and inflammation-related genes with AD [40,41,42] has also led to the proposal of the inflammation hypothesis. This hypothesis posits that neuroinflammation plays a primary role in the pathogenesis of AD, preceding rather than following Aβ deposition and tau pathology. It assumes that upon exposure to certain endogenous or external stimuli, activated microglia and reactive astrocytes produce and sustain a dysregulated neuroinflammatory response that acts as an upstream trigger of the complex pathological cascade that drives AD progression [43].

### 2.5. The Infection and Antimicrobial Protection Hypotheses

In line with the idea that AD pathophysiology has its roots in the activation of innate immune pathways, the infection hypothesis suggests that microbial agents and associated virulence factors may act as pathogenic triggers for AD onset [44,45,46], mainly through inflammatory processes [47]. This hypothesis has gained increasing relevance, supported by mounting evidence that Aβ may serve a protective role in the innate immune system, functioning as an AMP with activity against Gram-positive and Gram-negative bacteria, fungi [15,48], and viruses [49,50]. These findings, alongside data on harmful inflammatory pathways driving neurodegeneration, have led to a renewed infection hypothesis that reinterprets the amyloid cascade hypothesis within the framework of an innate immune response to microbial invasion—the antimicrobial protection hypothesis—which portrays Aβ deposition in the brain as a defence mechanism to counteract infections [16]. In this model, microbial challenge activates the innate immune system, fuelling a microglia-mediated neuroinflammatory response that reduces Aβ clearance [51] and induces expression of APP, BACE1, and γ-secretase in reactive astrocytes, resulting in increased Aβ concentration [52]. While at first Aβ production and oligomerisation may be beneficial to mediate pathogen entrapment/neutralisation and to restore brain homeostasis, chronic activation of this normally protective antimicrobial Aβ pathway leads to excessive Aβ accumulation and, ultimately, to senile plaque formation with subsequent tau phosphorylation. In addition, microbial agents may not only fuel neuroinflammation but also accumulate Aβ in the brain once their Aβ-containing biofilm constituents are integrated into plaque, thus contributing to plaque deposition. Plaque Aβ itself provides further stimulus for the innate immune system, enhancing the neuroinflammatory process in a progressive self-perpetuating amplifying circle of Aβ accumulation and sustained inflammation that, together with tau pathology, mediate neurodegeneration and AD progression. This process is influenced by genetic/epigenetic factors, stress, and age-related changes (e.g., immunosenescence, inflammageing, BBB impairment, etc.) that may exacerbate the inflammatory response to chronic infection [16,53].

Over the years, different pathogens have been suggested as potential drivers of AD pathogenesis [14] in a broad list that includes viruses [54], bacteria [55,56], fungi [57], as well as dysbiotic gut and oral microbiomes [58,59]. While probably more than one pathogen may be at play [20,44], *Pg* has been particularly implicated in the aetiology of AD [11,60]. This periodontal bacterium plays a crucial role in the pathogenesis of PeD, a chronic inflammatory disease linked to bacteraemia, chronic systemic inflammation, and several chronic inflammation-driven disorders, including AD [61]. PeD-associated systemic inflammation is suspected of promoting neuroinflammation and subsequent brain damage, either through cytokine-mediated microglial activation or by facilitating brain infection via a weakened BBB [14]. Within the antimicrobial protection hypothesis, which highlights chronic innate immune activation as a major driver of AD [16], a causative relationship between PeD and AD seems plausible. Thus, we propose that *Pg*, whose association with AD we will later summarise in this review, may represent a promising target for therapeutic or prophylactic interventions aimed at fighting AD.

## 3. Periodontitis and *Porphyromonas gingivalis*

PeD is a common chronic inflammatory oral disease of polymicrobial infectious aetiology, ranking 11th among the most prevalent conditions in the world according to the Global Burden of Disease Study (2016) [62]. Its global prevalence is estimated to range from 20 to 50% of the adult population [63], reaching up to 70% among adults aged 65 or older in the USA [64]. Clinically, PeD is characterised by progressive inflammatory destruction of the periodontium—the collective apparatus of specialised tissues surrounding and supporting the teeth (gingival complex, alveolar bone, periodontal ligament, and root cementum)—ultimately manifesting as attachment loss, alveolar bone resorption, bleeding on probing, and, eventually, tooth loss [65].

The pathogenesis of PeD involves the disruption of the delicate homeostatic balance between the commensal oral microbiota and the host immune surveillance, leading to the conversion of a symbiotic periodontal microbiota into a dysbiotic one that can trigger and sustain chronic, destructive inflammation [66]. Although the exact mechanisms driving this conversion are not fully understood, both host susceptibility and low-abundance keystone pathogen species are likely required to shift the balance toward dysbiosis [65], eventually precipitating tooth loss, systemic infection/inflammation, and subsequent systemic complications [65,67].

**Figure 3:**
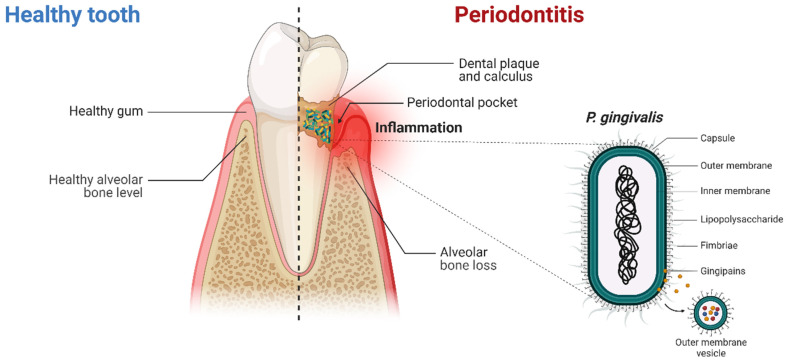
Schematic illustration of healthy and diseased periodontal tissues, highlighting *Pg* and its most significant virulence factors, a key contributor to periodontal dysbiosis and the progression of periodontitis.

Among the bacteria known to synergistically contribute to the pathogenesis of PeD [68], the most prominent species appears to be *Pg* (Figure 3), an asaccharolytic anaerobic Gram-negative bacterium that has long been linked to PeD [69]. *Pg* is a natural constituent of the oral microbiota; however, under certain conditions [70], it can colonise the subgingival plaque biofilm of healthy individuals and invade periodontal tissues, establishing a dysbiotic microbial community in the periodontal pocket [71]. Moreover, *Pg* evades host immune defences and modulates nutrient availability within the subgingival niche by eliciting inflammation, allowing for the continued expansion of dysbiotic consortia under persistent inflammatory conditions [71].

The capacity of *Pg* to evade host surveillance and sustain impaired inflammatory responses is determined by its repertoire of virulence factors [72], which most notably include: the outer membrane lipopolysaccharide (LPS), a potent activator of proinflammatory and immune responses primarily via Toll-like receptor (TLR) 2 and 4 signalling [73]; the gingipains, which are secretory or outer membrane-attached arginine- (RgpA, RgpB) and lysine-specific (Kgp) cysteine proteinases involved in tissue destruction, bacterial invasion of host tissues and systemic circulation, disruption of tight-junctions, complement inactivation through C3 and C5 degradation, and the breakdown of cytokines, TLR coreceptors, immunoglobulins, and AMPs [71]; the capsule, whose structural variability of its polysaccharides (K-antigens) defines bacterium serotype (K1–K7) and virulence capacity, protects the bacterium from opsonisation and phagocytosis by masking LPS and thus preventing complement activation [74]; the fimbriae, which mediate bacterial adhesion and invasion of host cells and tissues [75]; and the outer membrane vesicles (OMVs), which serve as vehicles for local and systemic dissemination of virulence factors such as LPS and gingipains [76]. Leveraging these and other virulence factors, *Pg* employs a wide range of strategies for immune evasion and suppression, host colonisation, tissue degradation, nutrition, and dissemination, ultimately perpetuating local periodontal disease and enabling bacterial access—via haematogenous, oro-pharyngeal, oro-olfactory, and oro-gastrointestinal routes—to distant organs, where it can induce or exacerbate inflammatory pathologies [61,77].

The following sections review the evidence linking PeD with AD, focusing primarily on studies associating PeD—or its keystone pathogen *Pg*—with AD. Nevertheless, it is important to note that other periodontopathic bacteria have also been implicated in the disease [78,79].

## 4. Human Epidemiological and Genetic Evidence Linking PeD/*Pg* with AD

Before tooth loss was first identified as a potential risk factor for AD [80], the association between oral health and AD merely resided on the idea that impaired cognition predisposes patients with dementia to various oral health issues, including PeD, due to poor oral hygiene, age-related or iatrogenic salivary flow dysfunction, and inability to seek professional dental care [81,82]. Since then, several studies have investigated the reverse relationship, linking oral disease and inadequate oral hygiene to an increased risk of cognitive decline, overall dementia, or AD-specific dementia [83,84,85]. For instance, a history of tooth loss before age 35 has been identified as a significant risk factor for AD, corroborating earlier findings [83]. A longitudinal study tracking 76 participants over 12 years further demonstrated that individuals with fewer teeth had a higher risk of developing dementia [84]. Subsequent studies investigating the association between the number of missing teeth and cognitive performance have reinforced the notion that tooth loss may be a predisposing factor for cognitive decline or dementia [86,87]. Although PeD is widely regarded as the primary cause of tooth loss among adults over 40 [88], studies focusing solely on associating tooth loss with cognitive outcomes do not provide conclusive evidence for linking PeD with these outcomes. This is largely because tooth loss can result from various other factors, including dental caries, orthodontic, prosthodontic, and endodontic treatments, trauma, and iatrogenic causes [89].

In the past decade, research has focused on the epidemiological association between direct measures or biomarkers of PeD and AD-related outcomes (Table 1), generally supporting the hypothesis that PeD per se may be associated with an increased risk of developing AD. In parallel, genetic-based studies have emerged, either applying advanced genetic epidemiological approaches to estimate the potential causal effect of PeD on AD risk [90] or employing bioinformatic analyses to investigate the genetic and molecular crosstalk mechanisms linking both diseases [12,91,92]. Table 2 summarises the evidence produced from such studies, which we further review in the following sections alongside the epidemiological data from Table 1.

### 4.1. Evidence Linking Clinical PeD with AD

Longitudinal evidence supporting a potential association between PeD and AD first emerged from a community-based prospective cohort study that followed 597 dentate men (28–70 years) for 32 years, ultimately concluding that PeD progression during follow-up independently increased the risk of incident cognitive decline in older men [93]. Subsequently, a 3-year [94] and, more recently, a 5-year cohort study [95] demonstrated that severe PeD was associated with incident cognitive impairments among community-dwelling elderly Japanese. Population-based data linked clinical PeD to a higher risk of developing dementia across 10-year retrospective cohorts comprising 8828 (≥20 years), 6056 (≥65 years), and 182,747 (≥45 years) participants [96,97,98]. Notably, treating PeD patients reduced their dementia risk [98], aligning with an earlier interventional pre–post study that found periodontal treatment could improve cognitive function in mild AD patients [99]. Another noteworthy finding from the aforesaid 10-year cohorts came from Tzeng’s study, which observed that the difference in cumulative dementia risk between PeD and non-PeD groups became significant only after 8 years of follow-up [96]. Closely consistent with this timeline, a retrospective cohort study of 27,963 individuals (≥50 years) found that PeD was associated with an increased risk of AD diagnosis only after 10 years of exposure [13]. Another yet larger (262,349 subjects) cohort study in which PeD patients were monitored for 10 years confirmed the observations from the previous studies, showing that patients with PeD were at higher risk for both overall dementia and AD [100]. More recently, the largest longitudinal study herein described (439,760 subjects) reported that over 4 years of follow-up, the incidence of AD-related dementias (ADRD) among HCV patients—a group already at increased dementia risk [101]—was higher and emerged earlier when PeD was also present, suggesting that multiple infections may synergistically accelerate ADRD onset [102]. Concerning cognition specifically, one prospective study found that gingival inflammation, but not clinical attachment level or probing pocket depth (PPD), was associated with incident cognitive decline in community-dwelling older adults [103]. Building on preliminary cross-sectional findings [104], a population-based study in Sweden prospectively linked history of PeD—reflected by radiographic alveolar bone loss—to cognitive decline in older adults [105]. Using comparable criteria, a case–control study produced similar results among elderly Koreans [106]. Likewise, other case–control and cross-sectional investigations, further detailed in Table 1, have generally reported associations between clinical measures of PeD and either cognitive decline [107,108,109], MCI/dementia [110,111,112], or AD per se [113,114,115,116]. Despite the overall trend supporting this relationship, some longitudinal studies have yielded either inconclusive [117] or contradictory findings [84,118,119], which is unsurprising given the lack of methodological homogeneity among epidemiologic studies, particularly when measuring or classifying both periodontal and cognitive status, a problem already identified by recent systematic reviews on the matter [120,121,122]. Moreover, all but two [84,119] of these discordant studies relied on cohorts with less than 10 years of follow-up, which—given the suggested ~10-year lag phase for newly diagnosed PeD to become a risk factor for AD [13,96,123]—may help to explain these conflicting findings. This temporal aspect may also clarify the mixed results of the Mendelian randomisation (MR) study by Sun et al., which did not consistently associate genetically predicted PeD with higher AD risk (or vice versa) [90]. As MR methods estimate average lifetime risks, they may not adequately capture how the timing and duration of PeD exposure influence AD risk [124]. Meanwhile, recent retrospective cohort studies with follow-up periods of 14 [125], 20 [126], and 26 years [127] have successfully associated PeD with an increased risk of incident overall dementia or AD, reinforcing the idea that long-term exposure to PeD may indeed be necessary for such associations to emerge [128].

### 4.2. Evidence Linking Immunological Markers of PeD with AD

As previously mentioned, PeD is suspected of challenging the brain primarily through bacterial invasion and immune–inflammatory mediators fuelled by recurrent, transient bacteraemia of oral origin that promotes a systemic proinflammatory state [14]. Strengthening this notion, evidence of a possible 10-year lag period for PeD to affect cognition bolsters the hypothesis that these ongoing peripheral immune–inflammatory responses may slowly and gradually drive neuroinflammation and subsequent neurodegeneration in AD. Underscoring this crosstalk as a possible mechanism by which PeD elevates AD risk, a recent transcriptomic analysis identified six core crosstalk genes linking PeD and AD that, notably, also connect the peripheral immune system with the central nervous system (CNS) [92]. In parallel, various case–control studies examining the blood of AD patients have implicated proinflammatory cytokines, including TNF-α [129,130,131] and IL-6 [131], in the overlapping mechanisms between PeD and AD. Moreover, a cohort investigation following 59 mild-to-moderate AD patients over 6 months revealed that those with concurrent PeD at baseline experienced both a faster rate of cognitive decline and a relative increase in the proinflammatory state compared to those without baseline PeD, thereby reinforcing the idea that PeD can actively fuel AD progression, likely via systemic inflammation [132]. An earlier case–control study had also found a similar association in relation to AD diagnosis, demonstrating that elevated plasma IgG against periodontal bacteria, including anti-*Pg* IgG—a reliable biomarker of periodontal infection [133]—combined with TNF-α levels could effectively distinguish clinical AD from cognitively normal individuals [129]. Indeed, growing immunological evidence indicates a relationship between heightened humoral responses to various periodontal pathogens and AD [79,123,127,134]. Increased antibody levels to *F. nucleatum* and *P. intermedia* were found in individuals who developed AD (versus cognitively intact controls) an average of 9.6 years before AD diagnosis [123], a timeframe that notably matches the 10-year lag period predicted by clinical markers of PeD [13,96]. Moreover, a larger longitudinal study with a 26-year follow-up period identified multiple associations between baseline serum IgG to periodontal pathogens—especially *Pg*—and both AD incidence and mortality [127]. This finding supports the notion that exposures of PeD, here represented as periodontal antibody reactivity, may precede and influence the onset of clinical AD. By contrast, a more recent 21-year cohort study failed to detect significant associations between clusters of IgG antibodies against periodontal microorganisms and AD mortality [135], suggesting that while PeD may be linked to AD incidence, its connection to AD mortality remains complex and requires further investigation.

### 4.3. Evidence Linking PeD with AD Pathology

Increasing evidence has demonstrated exposures of PeD to correlate with biomarkers of AD pathology, both in demented patients [111,136] and, more intriguingly, in cognitively healthy elderly [137,138]. A case–control study found an association between cerebrospinal fluid (CSF) total tau (t-tau)—a biomarker for brain tauopathy [139]—and serum anti-*Pg* antibody levels in AD patients [136]. More recently, a 2-year cohort study reported that PeD was associated with the progression of cognitive decline in elderly hypertensive individuals, highlighting a potential role for increased plasma levels of p-tau and Aβ_1–42_ in this effect [140]. Earlier research also linked clinical measures of severe PeD to elevated serum Aβ_1–42_ in mildly cognitively impaired or demented patients [111]. Combined with data revealing higher APP expression in PeD-affected gingival tissues [141], these findings suggest that PeD may enhance peripheral Aβ pathology, possibly via increased peripheral Aβ production [128]. Regarding central Aβ pathology, a cross-sectional analysis in cognitively healthy elderly revealed clinical measures of PeD to be associated with higher Aβ loads in brain areas usually prone to Aβ accumulation in AD [137]. More recently, another cross-sectional investigation in healthy elderly identified a correlation between increased periodontal bacterial dysbiosis and reduced CSF Aβ_42_ levels [138], a trend commonly considered a preclinical indicator of amyloidosis and emerging clinical AD [142]. Albeit merely correlational, these findings suggest an early involvement of PeD in AD pathology. To further investigate the role of PeD in preclinical AD, Schwahn et al. evaluated the impact of periodontal treatment on selected preclinical outcomes—AD score [143] and brain age gap [144]—through an innovative trial emulation approach based on observational data from 177 treated and 409 unintentionally untreated PeD patients. While the treatment effect on brain ageing was uncertain, a strong to moderate favourable effect was observed for AD score (an imaging biomarker for brain atrophy). Moreover, the identification of a dose–response relationship between PPD and AD score/brain age led the authors to conclude that PeD may be causally related to preclinical AD [145]. In contrast, a recent longitudinal study investigating the prospective association between PeD and markers of dementia and AD pathology, including brain volume and Aβ positivity, found no significant links after a follow-up period of more than 10 years [146]. This result complicates the debate over whether PeD exerts a causal influence on AD pathology during the earliest preclinical stages, highlighting the need for further clarification from future longitudinal and interventional research.

### 4.4. Presence of Pg and Periodontal Bacteria in AD Human Brains

Over the years, numerous studies have also focused on detecting and/or quantifying selected microorganisms, particularly periodontal bacteria, in relevant biological samples to identify meaningful associations with AD. Oral sampling, for instance, has revealed that exposures such as an increased load of periodontopathic bacteria in subgingival plaque [115], subgingival periodontal dysbiosis [138], and altered salivary [59,147,148] or supragingival dental biofilm microbiomes [149] are associated with AD-related outcomes. Even so, some of the most compelling evidence for the role of periodontal bacteria in the development of AD has come from post mortem studies investigating the presence of these bacteria and/or their virulent components in the brain tissue of AD patients. For example, *Treponema* species (including the well-known periodontopathic *T. denticola*) were identified more often in brain cortex samples from AD patients (14/16) compared to non-AD donors (4/18) [78], offering the first indication that periodontal anaerobes might reach the brain and contribute to AD pathogenesis. A subsequent post mortem study provided statistically significant evidence to implicate the presence of LPS from the keystone pathogen *Pg* in AD brains (4/10) versus controls (0/10) [150], further suggesting that a brain innate immune response against periodontal bacterial antigens may be involved in the mechanistic processes driving AD. Other investigations, however, have reported more equivocal findings on the prevalence of periodontal pathogens in AD brain specimens. Emery et al., for example, showed that while AD samples harboured larger overall bacterial loads than controls, they did not detect the periodontal pathogens most commonly linked to AD—though they acknowledged that these could be present at low copy numbers or in unexamined locations [56]. Likewise, Bennett et al. observed only borderline significance for higher frequencies of *Pg* and *Borrelia burgdorferi* in AD frontal cortex samples compared to controls, also noting that *Pg* was far from being the dominant species in either group [151]. Although these results appear to conflict with Poole’s earlier findings [150], they do not necessarily contradict the idea that, similar to its keystone pathogenic role in the subgingival biofilm, even small amounts of *Pg* could suffice to induce local inflammation in the brain, evade host surveillance, and ultimately help establish a thriving proinflammatory microbial community capable of triggering a persistent, destructive host response [152]. If so, this implies that the range of microorganisms found to be elevated in AD brain specimens or associated with AD pathology thus far [14] may actually access the brain and influence AD pathogenesis secondarily to a *Pg* brain infection—particularly once the BBB is compromised. Indeed, KEGG pathway enrichment analyses of overlapping genes between the *Pg* interactome and GWASdb AD-related genes highlighted pathways relevant to BBB function, including focal adhesion, junction, and actin signalling [12]. These findings align with those from a comparable analysis showing that PeD/AD core crosstalk genes are associated with overlapping functional terms related to focal adhesion and the extracellular matrix–receptor interaction pathway [91]. It is then reasonable to assume that the main effects of *Pg* may include BBB disruption—an early-occurring event in AD [153]—thereby paving the way for other pathogens to invade the brain. Furthermore, transcriptomic data show that misregulated genes in PeD and *Pg* microarrays match those misregulated in AD; however, less significantly for the bacterial transcriptome when compared with PeD. This difference suggests that other periodontal pathogens besides *Pg* may contribute to AD [12]. Meanwhile, the demonstration of biofilms colocalised with Aβ in the senile plaques of AD human brains [154], along with the confirmation that Aβ has antimicrobial properties [15], has led some authors to propose that these plaques might be foci of microbial biofilms [155,156], where multiple synergistic microbial species persist [53], despite innate immune efforts to counteract them, as reflected by the buildup of antimicrobial Aβ surrounding the biofilms [157]. Investigations of these biofilms have focused primarily on detecting *B. burgdorferi* [156,158]. As both are spirochetes, similar findings could apply to *T. denticola*, which, in addition to its associations with AD, is known to synergistically interact with *Pg* in forming subgingival biofilms during PeD pathogenesis [159]. Herpesviruses, arguably one of the pathogens more frequently correlated with AD and long ago identified within Aβ plaques [160], have also been shown to display a myriad of synergistic mechanisms with *Pg* and other periodontopathic bacteria in driving PeD pathogenesis [161]. It is thus reasonable to suggest that these and other putative pathogens may exhibit cooperative virulence within established brain biofilms, enabling them to evade host defences and sustain chronic infection and inflammation. If, as in the subgingival niche, the formation of pathogenic brain biofilms is also dictated by keystone pathogen species with the capacity to colonise the host, activate and evade immune responses, and promote group survival [67], then *Pg*—known as a “master evader” armed with an arsenal of virulence factors—emerges as the prime candidate. Although other evaders [158] or LPS-containing bacteria [55] are also known to infiltrate human brains and associate with AD pathology, only *Pg* is equipped with potent gingipains—specific toxic proteases adept at mediating host invasion, modulating inflammatory processes, and neutralising immune defences such as the complement cascade [162]. This endows *Pg* with a unique ability to not only elicit the pathological responses characteristic of AD but also subvert immune surveillance and sustain the survival of the biofilm community [163].

Strongly reinforcing the proposition that *Pg* and its gingipains are central players in the pathogenesis of AD, a seminal study by Dominy et al. demonstrated the presence of them both in post mortem brains of AD patients, with gingipains notably colocalising with neurones, astrocytes, tangles, and plaques in AD hippocampal specimens [11]. Furthermore, a positive association was found between gingipain brain levels and both AD diagnosis and pathology (tau and ubiquitin loads). Although specificity questions arose from the detection of *Pg* and gingipains in most non-demented controls, the authors noted the presence of AD pathology—and respective association with gingipain levels—even in these asymptomatic controls, suggesting preclinical AD. Therefore, when considering the directionality of events, this observation supports the notion that *Pg* does not access the brain as a consequence of symptomatic AD but may instead drive the pathological changes that precede clinical AD. Detection of *Pg* DNA in CSF samples from living AD patients provided additional evidence that the bacterium can access the CNS before death, alleviating reasonable concerns [164] about potential post mortem contamination of the aforementioned brain specimens. Shedding light on the potential for causation, Dominy et al. further conducted a series of preclinical studies, demonstrating not only that oral infection of BALB/c mice with *Pg* could lead to brain colonisation, neurodegeneration, and induction of the stereotypical AD marker Aβ_1–42_ but also that gingipains had neurodegenerative effects both in vivo and in vitro, exerting harmful impact on tau. More importantly, small-molecule gingipain inhibitors proved effective in both reversing experimental *Pg*-induced AD-like pathology and preventing gingipain-induced neurotoxicity [11], yielding the most compelling evidence to date implicating *Pg*-derived gingipains in AD pathogenesis.

**Table 1:** Human epidemiological studies on the association between periodontitis/*Pg* and Alzheimer’s disease.

Reference	Study Design	Main Objective	Sample Characterisation	Assessment	Findings
Kaye et al. (2010), USA [93]	Prospective cohort (32 y)	Determine whether rates of tooth loss, PeD progression, and caries incidence predict cognitive decline	597 subjects (28–70 y)	Exposures: > rate of tooth loss > PeD progression (PPD and ABL) > caries incidence Outcome studied: incident cognitive decline (MMSE, SCT)	Higher risk of low cognitive scores among >45-year-old subjects with higher rates of tooth loss, ABL progression, PPD progression, and new caries
Ide et al. (2016), UK [132]	Observational cohort (6 mo)	Examine the effect of PeD on the cognitive status and systemic proinflammatory status of AD patients	59 subjects (M_A_ = 77.7 y) > w mild-to-moderate AD and PeD (n = 22) > w mild-to-moderate AD only (n = 37)	Exposure: PeD (CDC/AAP def.) Outcomes studied: > variation in cognitive decline (NINCDS-ADRDA, ADAS-cog, and sMMSE) > variation in serum proinflammatory state (serum CRP, TNF-α, and IL-10)	Concurrent PeD at baseline was associated with increases in the proinflammatory state and rate of cognitive decline (6-fold, ADAS-cog)
Iwasaki et al. (2016), Japan [94]	Retrospective cohort (3 y)	Examine the relationship between severe PeD and cognitive decline	85 subjects (≥75 y, M_A_ = 79.3 y) > w severe PeD (n = 21) > wo severe PeD (n = 64)	Exposure: severe PeD (CDC/AAP def.) Outcome studied: incident cognitive decline (MMSE)	Severe PeD was associated with cognitive decline
Iwasaki et al. (2019), Japan [95]	Prospective cohort (5 y)	Determine the effect of severe PeD and periodontal inflammation (PI) on the incidence of MCI	179 subjects (≥75 y, M_A_ = 80.1 y) > w severe PeD > wo severe PeD	Exposures: > severe PeD (CDC/AAP and EWP defs.) > PI (PISA) Outcome studied: incident MCI (diff. diagnostic criteria)	Severe PeD and PI were associated with MCI
Tzeng et al. (2016), Taiwan [96]	Retrospective cohort (10 y)	Determine the effect of chronic gingivitis/PeD on the risk of dementia	8828 subjects (≥20 y) > w chronic gingivitis/PeD (n = 2207) > wo chronic gingivitis/PeD (n = 6621)	Exposure: chronic gingivitis/PeD (ICD-9-CM) Outcome studied: incident dementia (DSM-IV, DSM-IV-TR)	Chronic gingivitis/PeD patients with a higher risk for dementia
Lee YT et al. (2017), Taiwan [97]	Retrospective cohort (10 y)	Evaluate PeD as a modifiable risk factor for dementia	6056 subjects (≥65 y, M_A_ = 72.4 y) > w PeD (n = 3028) > wo PeD (n = 3028)	Exposure: PeD (ICD-9-CM) Outcome studied: incident dementia (ICD-9-CM)	PeD patients with a higher risk for dementia
Chen et al. (2017), Taiwan [13]	Retrospective cohort (16 y)	Determine the effect of PeD on the risk for AD	27,963 subjects (≥50 y, M_A_ = 54.2 y) > w PeD (n = 9291) > wo PeD (n = 18,672)	Exposure: PeD (ICD-9-CM) Outcome studied: incident AD (ICD-9-CM) > ≥1 y after exposure (excl. of diagnosed AD < 1 y) > ≥10 y after exposure (excl. of diagnosed AD < 10 y)	Only ≥ 10-year PeD-exposed patients with a higher risk for developing AD
Choi et al. (2019), South Korea [100]	Retrospective cohort (10 y)	Determine the effect of PeD on the risk for overall dementia, AD, and vascular dementia (VD)	262,349 subjects (≥50 y, M_A_ = 60.2 y) > w PeD (n = 46,344) > wo PeD (n = 216,005)	Exposure: PeD (ICD-10) Outcomes studied: incident overall dementia, AD, and VD (ICD-10)	PeD patients with a higher risk for overall dementia and AD
Nilsson et al. (2018), Sweden [105]	Prospective cohort (6 y)	Evaluate whether PeD is associated with cognitive decline	704 subjects (≥60 y) > w PeD > wo PeD	Exposure: history of PeD (radiographic ABL-based criterion as indicator) Outcome studied: incident cognitive decline (MMSE)	History of PeD was associated with cognitive decline
Kim et al. (2020), South Korea [125]	Retrospective cohort (14 y)	Evaluate severe PeD with tooth loss as a modifiable risk factor for AD, VD, and mixed dementia (MD)	20 230 subjects (40–79 y) > w severe PeD with tooth loss (n = 10,115) > wo severe PeD with tooth loss (n = 10,115)	Exposure: severe PeD (defined as requiring surgical intervention) with tooth loss Outcomes studied: incident AD, VD, and MD (KCD-7)	Severe PeD with 1–9 remaining teeth at higher risk of AD, VD, and MD
Demmer et al. (2020), USA [126]	Retrospective cohort (20 y)	Evaluate whether PeD is associated with increased risk for dementia and MCI	8275 subjects (M_A_ = 63 y) > w PeD > wo PeD	Exposure: PeD (PPC, CDC/APP, and others) Outcomes studied: incident dementia and MCI/dementia composite (NINCDS-ADRDA and DSM-V)	Several associations only between the most diseased PPC categories and increased risk for incident dementia and incident MCI/dementia
Nikitin et al. (2021), USA [103]	Prospective cohort (11 y)	Assess the association between clinical measures of PeD and subsequent cognitive decline	967 subjects (70–79 y)	Exposure: PeD (GI, CAL, and PPD as indicators) Outcome studied: incident cognitive decline (3MS and DSST)	Only GI was consistently associated with higher cognitive decline
Malone et al. (2022), USA [102]	Retrospective cohort (4 y)	Examine whether PeD increases the risk of developing AD and related dementias (ADRD) among HCV patients	439 760 subjects with HCV (44% ≥65 y) > HCV patients w PeD (n = 32,231) > HCV patients wo PeD (n = 407,529)	Exposure: PeD (ICD-9 and ICD-10) Outcome studied: incident ADRD (MBSF data)	PeD increased the risk for ADRD among HCV patients
Holmer et al. (2022), Sweden [118]	Retrospective cohort (mean 7.5 y)	Determine the effect of deep PPD, as a PeD proxy, on the incidence of dementia	37 174 subjects (≥40 y, M_A_ = 61 y) > w deep PPD (n = 7992) > wo deep PPD (n = 29,182)	Exposure: PeD (deep PPD as proxy) Outcome studied: incident dementia (ICD-10, others)	Deep PPD was not associated with dementia
Adam et al. (2022), USA [146]	Prospective cohort (max. 17 y)	Investigate whether PeD is prospectively associated with cerebrovascular and neurodegenerative markers of dementia and AD pathology	1306 subjects for MRI cohort (M_A_ = 61.8 y) 248 subjects for PET cohort (M_A_ = 61.2 y) > w PeD > wo PeD	Exposure: PeD (modified PPC) Outcomes studied: > altered brain volumes (MRI) > incident microhaemorrhages (MRI) > incident brain β-amyloid positivity (PET scan)	PeD was not associated with altered brain volumes, microhaemorrhages, or β-amyloid positivity
Carballo et al. (2023), Spain [140]	Prospective cohort (2 y)	Evaluate whether PeD is associated with cognitive decline and blood-based markers of AD	101 subjects with a history of hypertension (≥60 y, M_A_ = 71 y) > w PeD (n = 63) > wo PeD (n = 38)	Exposure: PeD (CDC/AAP def.) Outcomes studied: > variation in cognitive decline (MMSE and ACE) > variation in plasma levels of Aβ_1–40_, Aβ_1–42_, p-tau, and t-tau	PeD was associated with cognitive decline, its progression, and increased plasma levels of Aβ_1–40_ and p-tau
Lee YL et al. (2017), Taiwan [98]	Retrospective cohort (10 y)	Assess the effect of PeD severity and PeD-related treatments on the incidence of dementia	182,747 subjects with PeD (≥45 y) assigned to: > dental prophylaxis (n = 97,802) > PeD-intensive treatment (n = 5373) > PeD w tooth extraction (n = 59,898) > PeD wo treatment (n = 19,674)	Exposure: one of the four treatments (treatment received as a marker for PeD severity) Outcome studied: incident dementia (ICD-9-CM)	Higher risk of dementia for patients with untreated or more-severe PeD (tooth extraction) PeD prevention or treatment might reduce or delay the development of dementia
Schwahn et al. (2022), Germany [145]	Quasi-experimental—trial emulation approach (21 y)	Investigate the relationship between PeD treatment and the preclinical AD	586 subjects with criteria for periodontal treatment (<60 y, M_A_ = 45 y) > treated from GANI_MED (n = 177) > untreated from SHIP-TREND (n = 409)	Exposure: periodontal treatment Outcomes studied: > AD score [143] (brain atrophy MRI marker) > brain age gap [144] (MRI)	Periodontal treatment had a favourable effect on the preclinical AD-related AD score
De Souza Rolim et al. (2014), Brazil [99]	Interventional pre–post (6 mo)	Compare the orofacial characteristics and functional/cognitive aspects of AD patients before and after dental treatment	29 subjects with mild AD (age not specified) > no control group	Exposure: dental treatments (mainly periodontal treatment) Outcomes studied: > cognitive deficit (MMSE) > functional cognitive impairment (Pfeffer’s questionnaire [165]) > orofacial characteristics (diff. clinical parameters)	At last evaluation after dental treatment (6 mo): > overall improvement of orofacial co-morbidities > improvement in MMSE and functional cognitive impairment
Beydoun et al. (2020), USA [127]	Retrospective cohort (26 y)	Examine the association between PeD pathogens and incident all-cause/AD dementia, and AD mortality	6650 subjects (≥45 y) from NHANES III, both phases 1988–94 3479 subjects (≥45 y) from NHANES III, phase 2 1991–94	Exposures: > serum IgG to PeD bacteria at baseline > PPD > CAL Outcomes studied: > incident all-cause and AD dementia (ICD-9) > AD mortality (ICD-10)	Multiple associations between bacterial IgG titers, particularly *Pg*, and AD incidence/mortality PPD was associated with incident AD
Merchant et al. (2024), USA [135]	Prospective cohort (21 y)	Examine associations between clusters of serum IgG to PeD bacteria and AD mortality	8153 subjects (≥40 y) from NHANES III	Exposure: serum IgG clusters to PeD bacteria at baseline Outcome studied: AD mortality	Clusters of serum IgG to PeD bacteria were not significantly associated with AD mortality
Sparks Stein et al. (2012), USA [123]	Nested case–control (mean 12.5 y for controls)	Compare baseline serum IgG levels to PeD bacteria between subjects who converted or not to AD during the follow-up	From 158 subjects (M_A_ = 71.5 y) cognitively intact at baseline serum draw: > cases: w incident AD (n = 35) or MCI (n = 46) > control: cognitively healthy at last follow-up (n = 77)	Exposure studied: serum IgG to PeD bacteria at baseline (as PeD proxy) Outcomes: > AD (NINCDS-ADRDA and MMSE) > MCI (Petersen’s criteria [166,167])	Increased anti-*Fn* and -*Pi* titers at baseline in the patients with incident AD
Noble et al. (2014), USA [79]	Case–cohort (mean 5 y)	Assess pre-morbid levels of serum IgG to PeD bacteria as possible predictors of incident AD	From 219 subjects (>65 y, M_A_ = 75.6 y) cognitively intact at baseline serum draw: > cases: w incident AD (n = 110) > control: cognitively healthy at last follow-up (n = 109)	Exposure studied: serum IgG to PeD bacteria at baseline (as PeD proxy) Outcome: AD (diff. diagnostic criteria)	Increased anti-*An* titer at baseline was associated with higher risk of AD Serum IgG levels to common PeD bacteria may be predictors of incident AD
Kamer et al. (2009), USA [129]	Case–control	Determine whether elevated cytokine expression and plasma IgG levels to PeD bacteria are associated with AD	24 subjects (≥40 y) > cases: w AD (n = 18) > control: cognitively healthy (n = 16)	Variables studied: > plasma IgG to PeD bacteria (as PeD proxy) > plasma TNF-α, IL-1β, and IL-6 (as systemic inflammation proxy) Outcome: AD (NINCDS-ADRDA, DSM-IV, and MMSE)	TNF-α levels and elevated numbers of IgG against PeD bacteria (*Pg*, *Aa*, and *Tf*) were associated with AD
Farhad et al. (2014), Iran [130]	Case–control	Evaluate the effect of PeD on serum levels of TNF-α in AD patients	80 subjects with AD (40–70 y) > cases: w PeD (n = 40) > control: wo PeD (n = 40)	Variable studied: serum TNF-α Outcome: AD with concomitant PeD (dental examination, CAL)	Serum TNF-α levels were 3-fold higher in the AD patients with concomitant PeD
Cestari et al. (2016), Brazil [131]	Case–control	Investigate the prevalence of oral infections and serum IL-1β, IL-6, and TNF-α in patients with MCI or AD	65 subjects (M_A_ = 75.6 y) > cases: w MCI (n = 19) or AD (n = 25) > control: cognitively healthy (n = 21)	Variables studied: > serum levels of IL-1β, IL-6, and TNF-α > PeD (diff. clinical parameters) Outcomes: MCI and AD (NINCDS-ADRDA and MMSE)	Elevated IL-6 in AD/MCI patients was associated with high serum TNF-α in PeD patients
Laugisch et al. (2018), Germany [136]	Case–control	Investigate the presence of PeD pathogens and intrathecal pathogen-specific IgG in AD and non-AD demented patients	40 subjects (30–70 y, M_A_ = 59.7 y) > cases: w AD (n = 20) > control: non-AD demented (n = 20)	Variables studied: > periodontal destruction (number of teeth, PPD, CAL, BoP) > presence of PeD bacteria in periodontium, serum, and CSF > periodontium and serum levels of IL-1β and MCP1/CCL2 > serum and CSF IgG to PeD bacteria > CSF levels of Aβ_1–42_ and t-tau Outcome: AD (NIA-AA 2011 and MMSE)	Subjects with AD presented: > no differences in the control of periodontal destruction, IgG to PeD bacteria, and cytokine levels > presence of PeD bacteria only in periodontium > higher IgG to PeD bacteria in CSF than in serum > lower Aβ_1–42_ and higher t-tau levels in the CSF > association of CSF levels of t-tau with both serum levels of anti-*Pg* IgG and MCP1/CCL2 Local production of IgG to PeD bacteria in the CSF may occur in demented patients, but there was no association with AD
De Souza Rolim et al. (2014), Brazil [113]	Case–control	Compare the oral status, mandibular function, and orofacial pain between mild AD patients and healthy subjects	59 subjects (59–91 y, M_A_ = 68 y) > cases: w mild AD (n = 29) > control: wo AD (n = 30)	Variables studied: PeD (diff. clinical parameters), others Outcome: mild AD (NINCDS-ADRDA and MMSE)	Higher prevalence of PeD in subjects with AD than in healthy subjects
Gil-Montoya et al. (2015), Spain [110]	Case–control	Determine whether PeD is associated with the diagnosis of cognitive impairment/ dementia	409 subjects (>50 y) > cases: w MCI/dementia (n = 180) > control: cognitively healthy (n = 229)	Variables studied: PeD (diff. clinical parameters) Outcomes: MCI/dementia (NINCDS-ADRDA, DSM-IV, and Robles et al. criteria [168])	Associations were found between clinical PeD parameters (particularly CAL) and cognitive impairment/dementia
Gil-Montoya et al. (2017), Spain [111]	Case–control	Determine whether PeD relates with serum Aβ load and assess the role of such relationship in the association between Aβ and cognitive impairment/dementia	288 subjects (M_A_ = 76.6 y) > cases: w MCI/dementia (n = 166) > control: cognitively healthy (n = 122)	Variables studied: > serum levels of Aβ peptides > PeD (CAL-based criterion as severity indicator) Outcomes: MCI/dementia (NINCDS-ADRDA, DSM-IV-TR, and Robles et al. criteria [168])	Severe PeD was associated with higher serum Aβ_1–42_ levels Serum Aβ_1–42_ levels were positively associated with cognitive impairment/dementia only in the severe PeD group
Shin et al. (2016), South Korea [106]	Case–control	Investigate the association between PeD and cognitive impairment	189 subjects (≥60 y, M_A_ = 69 y) > cases: w cognitive impairment (n = 65) > control: cognitively healthy (n = 124)	Variable studied: history of PeD (radiographic ABL) Outcome: cognitive impairment (MMSE-KC)	Subjects with a history of PeD were more likely to have cognitive impairment
Holmer et al. (2018), Sweden [112]	Case–control	Evaluate whether PeD increases the risk of MCI, subjective cognitive decline (SCD), and AD	230 subjects (50–80 y, M_A_ = 67.2 y) > cases: w MCI (n = 51), SCD (n = 51) or AD (n = 52) > control: cognitively healthy (n = 76)	Variables studied: PeD (marginal ABL, PPD, BoP, and others) Outcomes: MCI, SCD, and AD (ICD-10 and others)	Marginal PeD (generalised marginal ABL and increased PPD) was associated with the cases groups combined (MCI, SCD, and AD)
De Oliveira Araújo et al. (2021), Brazil [114]	Case–control	Determine whether PeD is associated with AD and its impact on the OHR-QoL perception	102 subjects (n = 50, M_A_ = 71.2 y) > cases: w mild-to-moderate AD (n = 50) > control: cognitively healthy (n = 52)	Variables studied: > PeD (PPD, CAL, BoP, others) > OHR-QoL (GOHAI) Outcome: mild-to-moderate AD (DSM, MMSE)	PeD was associated with AD, but not with OHR-QoL
Panzarella et al. (2022), Italy [115]	Case–control	Evaluate the relationship between measures of oral health and both amnestic MCI or AD	60 subjects (M_A_ = 80.0 y) > cases: w AD (n = 20) or aMCI (n = 20) > control: cognitively healthy (n = 20)	Variables studied: > dental status (DMFT score) > periodontal status (CPI and PSR scores) > subgingival plaque bacterial load > OHR-QoL (OHIP-14) Outcomes: > AD (NIA-AA 2011) > aMCI (modified Petersen’s criteria [167])	Subjects with AD showed: > poor health status related to PeD > higher DMFT scores than aMCI and control > higher *Fn* bacterial load than aMCI and control > no statistically significant differences in OHR-QoL
Poole et al. (2013), UK [150]	Case–control	Establish a link between PeD and AD by identifying the major PeD bacteria and/or bacterial components in post mortem human brain specimens	20 human post mortem (PM) brain tissue samples > cases: w AD diagnosis (n = 10) > control: wo AD diagnosis (n = 10)	Variables studied: > presence of major PeD bacteria (*Td*, *Tf*) in brain tissue > presence of *Pg*-LPS and/or *Pg* gingipains in brain tissue Outcome: AD	Statistically significant evidence was found to implicate the presence of *Pg*-LPS in AD cases
Dominy et al. (2019), USA [11]	Case–control	Demonstrate the presence of *Pg* DNA and gingipains in the brain of AD patients	Human post mortem (PM) brain tissue samples (n variable) > cases: w AD diagnosis > control: non-demented	Variables studied: > gingipains, tau, and ubiquitin load in brain tissue > presence of *Pg* DNA in brain tissue Outcome: AD	Gingipains load in the brain was correlated with AD diagnosis and pathology (tau and ubiquitin) RgpB gingipain colocalised with neurones, astrocytes, tau tangles, and intracellular Aβ in AD hippocampus *Pg* DNA and Kgp gingipain were identified in the AD cerebral cortex
	Prospective pilot	Demonstrate the presence of *Pg* DNA in the CSF of living subjects diagnosed with probable AD	10 CSF samples from subjects with probable AD (53–72 y)	Variable studied: presence of *Pg* DNA in CSF	*Pg* DNA was identified and quantified in the CSF of clinical AD patients
Liu et al. (2019), China [59]	Case–control	Compare the composition of oral microbiota between AD patients and healthy subjects	78 subjects (M_A_ = 64 y) > cases: w mild (n = 13), moderate (n = 12) or severe AD (n = 14) > control: cognitively healthy (n = 39)	Variable studied: composition of salivary microbiota Outcome: AD (NINCDS-ADRDA and MMSE)	Although no particular bacteria were associated with AD severity, the richness and diversity of salivary microbiota flora were significantly reduced in AD patients
Leblhuber et al. (2020), Austria [147]	Cross-sectional	Investigate whether the presence of PeD pathogens in saliva is associated with cognitive impairment in patients with probable AD	20 subjects with probable AD (M_A_ = 78.1 y)	PeD-related measure: presence of PeD pathogens (saliva) AD-related outcomes: > cognitive tests (MMSE and CDT) > serum levels of neopterin, tryptophan, and kynurenine	Salivary presence of *Pg* was associated with lower MMSE and CDT scores Salivary presence of *Td* and *Tf* were associated with lower neopterin and kynurenine serum levels, respectively
Noble et al. (2009), USA [134]	Cross-sectional	Investigate the relationship between systemic exposure to PeD pathogens and cognitive test outcomes	2355 subjects (≥60 y, M_A_ = 70.8 y) from NHANES III	PeD-related measure: serum levels of anti-*Pg* IgG AD-related outcomes: cognitive tests (for verbal memory and subtraction)	Subjects with the highest anti-*Pg* IgG levels were associated with poor delayed verbal memory and impaired subtraction
Kamer et al. (2012), Denmark [107]	Cross-sectional	Assess the effect of periodontal inflammation (PI) or tooth loss on cognitive functioning	152 subjects (70 y)	PeD-related measures: > PI (MCPI score) > tooth loss AD-related outcomes: cognitive tests (DSST and BDT)	The association of PI with DSST and BDT scores was dependent on the number of missing teeth Subjects with PI had lower adjusted mean DSST and BDT scores
Nilsson et al. (2018), Sweden [104]	Cross-sectional	Investigate the association between PeD or the number of teeth and cognitive functioning	775 subjects (60–99 y)	PeD-related measures: > extent of ABL > prevalence of PeD pockets ≥5 mm on ≥30% of teeth > number of teeth AD-related outcomes: cognitive tests (MMSE and CDT)	Loss of alveolar bone and lower number of teeth were statistically associated with a lower outcome on the MMSE test
Zhang et al. (2020), China [108]	Cross-sectional	Examine the relationship between poor oral health conditions and cognitive decline	102 subjects (52–101 y)	PeD-related measures: number of missing index teeth, BoP, and PPD AD-related outcome: cognitive test (MMSE)	Higher number of missing index teeth and higher average PPD were associated with lower cognitive MMSE scores
Marruganti et al. (2023), Spain [109]	Cross-sectional	Investigate the association between PeD and low cognitive performance	2086 subjects (≥60 y, M_A_ = 68.6 y) from NHANES 2011–2014	PeD-related measures: PPD and CAL AD-related outcomes: cognitive tests (CERAD-WL, AFT, and DSST)	Severe or moderate PeD was associated with low DSST PPD and CAL were associated with low global cognition performance
Kamer et al. (2015), USA [137]	Cross-sectional	Investigate the association between PeD and Aβ brain load	38 cognitively healthy subjects (44–79 y, M_A_ = 61.3 y)	PeD-related measures: > CAL (primary exposure) > number of teeth, dental plaque, BoP, PPD AD-related outcome: brain Aβ load in AD-vulnerable areas (PIB-PET scan)	Clinical measures of PeD were associated with Aβ accumulation in AD-vulnerable brain areas
Kamer et al. (2021), USA [138]	Cross-sectional	Investigate whether periodontal dysbiosis is associated with CSF markers of AD pathology	48 cognitively healthy subjects (M_A_ = 69.2 y)	PeD-related measures: > dysbiotic index (DI—primary exposure) > subgingival bacterial species cluster (secondary exposure) AD-related outcomes: CSF levels of Aβ_42_ and p-tau181	Higher periodontal dysbiosis was associated only with reduced CSF Aβ_42_
Schwahn et al. (2022), Germany [145]	Cross-sectional	Investigate the association between PeD and preclinical AD	1323 subjects (<60 y)	PeD-related measures: PPD, CAL, dental plaque, and calculus AD-related outcomes: > AD score [143] (brain atrophy MRI marker) > brain age (MRI)	Severe or moderate PeD involved in the continuum of preclinical AD severity: > dose–response relationship between PPD and AD score/brain age
Tiisanoja et al. (2019), Finland [116]	Cross-sectional	Investigate whether oral diseases and the related inflammatory burden are associated with diagnosed AD or dementia	170 subjects (≥75 y, M_A_ = 80.9 y)	Oral disease-related measures: > dental caries (number of carious teeth) > PeD (number of teeth with PeD pockets ≥4 mm) > stomatitis (visual inspection) > inflammatory burden (numeric score) AD-related outcomes: > diagnosed AD (DSM-IV) > all diagnosed dementias (DSM-IV and McKeith et al. criteria [169])	Dental caries and inflammatory burden were associated with a higher likelihood of having AD Subjects with PeD and stomatitis had an increased, although not statistically significant, likelihood of having AD

Abbreviations: 3MS, modified MMSE; *Aa*, *Aggregatibacter actinomycetemcomitans*; Aβ, amyloid beta; ABL, alveolar bone loss; ACE, Addenbrooke’s Cognitive Examination; AD, Alzheimer’s disease; ADAS-cog, Alzheimer’s disease assessment scale—cognitive subscale; ADRD, Alzheimer’s disease-related dementias; AFT, animal fluency test; aMCI, amnestic mild cognitive impairment; *An*, *Actinomyces naeslundii*; BDT, block design test; BoP, bleeding on probing; CAL, clinical attachment level; CDC/AAP, Centers for Disease Control and Prevention/American Academy of Periodontology; CDT, clock drawing test; CERAD-WL, Consortium to Establish a Registry for Alzheimer’s Disease Word Learning test; CPI, community periodontal index; CRP, C-reactive protein; CSF, cerebrospinal fluid; def., definition; DI, dysbiotic index; diff., different; DMFT, decayed, missing, and filled teeth; DNA, deoxyribonucleic acid; DSM-IV, Diagnostic and Statistical Manual of Mental Disorders, Fourth Edition; DSM-IV-TR, Diagnostic and Statistical Manual of Mental Disorders, Fourth Edition, Text Revision; DSM-V, Diagnostic and Statistical Manual of Mental Disorders, Fifth Edition; DSST, digit symbol substitution test; EWP, European Workshop in Periodontology Group C; excl., exclusion; *Fn*, *Fusobacterium nucleatum*; GI, gingival index; GOHAI, geriatric oral health assessment index; HCV, hepatitis C virus; ICD-10, International Classification of Diseases, 10th Revision; ICD-9, International Classification of Diseases, 9th Revision; ICD-9-CM, International Classification of Diseases, 9th Revision, Clinical Modification; IgG, immunoglobulin G; IL, interleukin; KCD-7, Korean Standard Classification of Diseases, 7th Revision; Kgp, lysine-specific gingipain; M_A_, mean age; max., maximum; MBSF, Medicare master beneficiary summary files; MCI, mild cognitive impairment; MCP1/CCL2, monocyte chemoattractant protein 1; MCPI, modified community periodontal index; MD, mixed dementia; MMSE, mini-mental state examination; MMSE-KC, Korean version of MMSE; mo, month(s); MRI, magnetic resonance imaging; n, number of subjects; NHANES, National Health and Nutrition Examination Survey; NIA-AA 2011, National Institute on Aging—Alzheimer’s Association guidelines published in 2011; NINCDS-ADRDA, National Institute of Neurological and Communicative Disorders and Stroke—Alzheimer’s Disease and Related Disorders Association; OHIP-14, oral health impact profile-14; OHR-QoL, oral health-related quality of life; PeD, periodontitis, periodontal; *Pg*, *Porphyromonas gingivalis*; *Pg*-LPS, *Porphyromonas gingivalis* lipopolysaccharide; PI, periodontal inflammation; *Pi*, *Prevotella intermedia*; PIB-PET, Pittsburgh compound B in positron emission tomography; PISA, periodontal inflamed surface area; PM, post mortem; PPC, periodontal profile class; PPD, probing pocket depth; PSR, periodontal screening and recording; p-tau, phosphorylated tau; RgpB, arginine-specific gingipain B; SCD, subjective cognitive decline; SCT, spatial copying task; sMMSE, standardised MMSE; *Td*, *Treponema denticola*; *Tf*, *Tannerella forsythia*; TNF-α, tumour necrosis factor alpha; t-tau, total tau; VD, vascular dementia; w, with; wo, without; y, year(s).

**Table 2:** Genetic evidence on the association between periodontitis/*Pg* and Alzheimer’s disease.

Reference	Study Characterisation	Findings
Jiang et al. (2021) [91]	**Bioinformatics study** Functional enrichment (GO and KEGG pathway) and PPI network analyses of the crosstalk genes between: > DEGs of AD gene expression datasets (from GEO) > PeD gene set (from text mining)	Identification/characterisation of shared molecular linkages (core crosstalk genes, GO and KEGG overlapping functional terms) between PeD and AD
Jin et al. (2021) [92]	**Bioinformatics study** Series of bioinformatic analysis of the crosstalk genes between: > AD-related genes (from DisGeNET) > DEGs of PeD gene expression datasets (from GEO) Identification of core crosstalk genes by further overlapping: > feature selected crosstalk genes > with PeD-related genes (from DisGeNET)	Identification of shared molecular linkages (core crosstalk genes, transcription factors, and pathways) between PeD and AD
Sun et al. (2020) [90]	**Bidirectional two-sample Mendelian randomisation** Analysis of genetically predicted PeD on the risk of AD: > GWAS of PeD: 4.924 cases vs 7.301 controls (5 SNPs or 7 SNPs) > GWAS of AD: 21.982 cases vs 41.944 controls	Higher risk of AD (only if using five SNPs as instruments)
Carter et al. (2017) [12]	**Bioinformatics study** Analysis of gene/environment interactions between PeD/*Pg* and AD-associated genes, by comparing: > *Pg*/host interactome > with GWAS AD susceptibility genes AND > microarray data from PeD tissue or *Pg*-treated macrophages > with microarray datasets from AD hippocampus	> *Pg*/host interactome highly enriched in susceptibility genes of AD > KEGG analysis revealed pathways relevant to BBB and inflammation AND > misregulated genes in PeD/*Pg* microarrays matched those in the AD hippocampus > overlaps less significant in the *Pg* microarrays

Abbreviations: AD, Alzheimer’s disease; BBB, blood–brain barrier; DEGs, differentially expressed genes; GEO, Gene Expression Omnibus; GO, gene ontology; GWAS, genome-wide association studies; KEGG, Kyoto Encyclopedia of Genes and Genomes; PeD, periodontitis; *Pg*, *Porphyromonas gingivalis*; PPI, protein–protein interaction; SNPs, single-nucleotide polymorphisms.

## 5. Opportunity for a Mucosal *Pg* Nanovaccine?

Based on the extensive data connecting both diseases, the model by which PeD may drive or contribute to AD pathogenesis reflects the effect of two potential main pathological mechanisms [128].

In the first mechanism, periodontal pathogens, their virulence factors, and immune–inflammatory mediators (e.g., cytokines and chemokines) in the periodontal milieu may enter the bloodstream, imposing a systemic inflammatory burden that can be further propagated to the brain through several documented processes [170,171]. Leptomeningeal cells, for instance, have been shown to transduce PeD-derived systemic inflammatory signals from peripheral macrophages to brain-resident microglia [172]. In addition, circulating bacterial products, specifically *Pg*-LPS, can directly activate leptomeninges and cerebral endothelial cells via TLR2 and TLR4 signalling to produce additional proinflammatory mediators that may be subsequently amplified by microglia, resulting in increased neuroinflammation [172,173].

The second mechanism reflects the possibility of periodontal pathogens, particularly *Pg* and/or their virulence factors, reaching the brain either directly through peripheral nerves (e.g., trigeminal or olfactory nerves) or via the bloodstream after spreading from the periodontal pocket or gastrointestinal tract [14,174]. Brain access through systemic circulation may involve the following pathways: (1) via perivascular spaces or special areas devoid of the BBB (e.g., circumventricular organs and choroid plexus) [175]; (2) via direct or phagocyte-assisted transcellular/paracellular crossing of a weakened BBB [176], whose function may be compromised by different inflammation- and *Pg*-mediated pathological processes [177]; and/or (3) via nanosized OMVs transporting key virulence factors across the BBB [178,179] and blood–CSF barrier [180].

In the brain, persistent exposure to *Pg* and/or its components, as well as to peripherally derived cytokines, can generate a chronic, microglia-mediated neuroinflammatory response [181,182,183,184,185,186], also promoting BBB permeability [173,187,188,189], Aβ production [190,191,192,193], and tau hyperphosphorylation [171,194,195,196]. Gingipains from *Pg* can directly fragment tau protein, adding to the latter pathway to enhance NFT formation [11]. Moreover, *Pg* may also reduce Aβ clearance [197], providing an additional mechanism to elevate brain levels of antimicrobial Aβ that can then aggregate with *Pg*-induced biofilms to form the characteristic senile plaques [53]. Beyond these central effects, *Pg* can enhance peripheral production of Aβ [198,199] and synergistically promote its influx into the brain [200], further increasing toxic Aβ brain accumulation and fuelling the ongoing inflammatory process with additional stimuli [201]. Alongside Aβ and tau proteinopathies, the release of inflammatory and oxidative mediators can damage neurones and synapses, generating the neurodegenerative changes that eventually lead to symptomatic AD [128,181]. Finally, PeD can induce gut dysbiosis [202], which may exacerbate low-grade systemic inflammation and BBB/gut barrier permeability—especially in advanced age—thus enabling additional cytokines, pathogens, and microbial products to spread and subtly influence AD pathogenesis [203].

**Figure 4:**
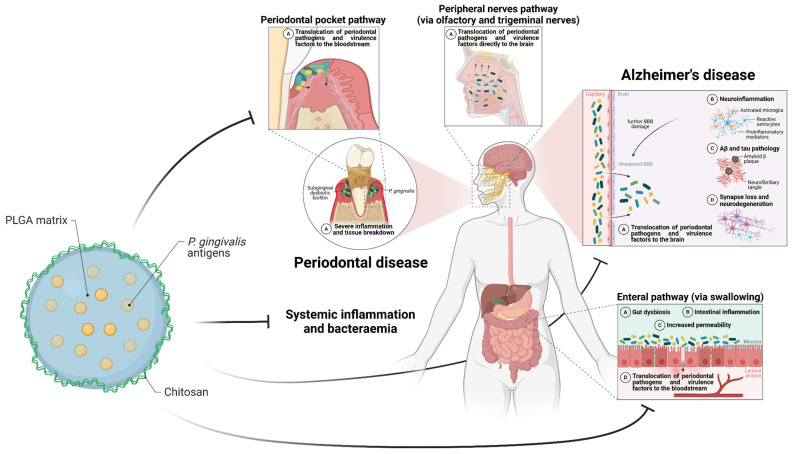
Model for the potential aetiological link between periodontitis/*Pg* and Alzheimer’s disease, highlighting the specific pathways targeted by the proposed mucosal nanovaccine to counteract *Pg*.

The recognition and clearer understanding of the mechanisms linking PeD and AD have provided a new conceptual framework for treating and preventing AD. Panza et al. recently advocated for clinical trials testing antibiotic therapy in AD patients exposed to suspected pathogens [14], while Dominy et al. proposed that brain-penetrant gingipain inhibitors could be more effective in reducing *Pg* brain infection and slowing or preventing disease progression without contributing to selective pressures for antibiotic resistance [11]. However, the simplest and broadest interventions may revolve around preventing AD by targeting the periodontal microbial aetiology before the clinical onset of the disease [204]. While treating PeD in its early stages or preventing it through diligent dental care and hygiene seem like straightforward approaches that could help prevent the dissemination of *Pg* and symbiotic bacteria into the CNS and potentially reduce AD incidence [205,206], future preventive strategies for AD could also involve vaccination against *Pg*, as suggested elsewhere [204,206,207]. Although vaccines proposed so far against this bacterium have focused solely on preventing PeD [208,209], the suggested mechanisms by which *Pg* spreads systemically and invades the brain present an opportunity to develop an effective prophylactic vaccine against AD. In support of this strategy, we propose that the aetiological link between PeD and AD could be broken by designing an oral nanovaccine containing *Pg*-specific antigens for mucosal delivery (Figure 4). Targeting the gut-associated lymphoid tissue (GALT), oral vaccination would elicit both mucosal and systemic immunity [210], thereby hindering *Pg*’s ability to cross the oral/intestinal barriers and the BBB, respectively.

Nanovaccine technologies have emerged as transformative approaches in prophylactic and therapeutic immunisation strategies, leveraging advancements in nanocarrier systems to enhance antigen delivery, immune activation, and tissue-specific targeting [211]. Among these, nanoparticulate vaccines based on the functional and biodegradable/biocompatible properties of polymers such as polylactic acid (PLA), poly(lactic-co-glycolic acid) (PLGA), and poly-ε-caprolactone (PCL), as well as natural polysaccharides like alginate and chitosan, have been extensively studied for a variety of diseases, demonstrating their capacity to enhance antigen stability, improve delivery to antigen-presenting cells (APCs), and stimulate protective humoral and cellular immunity [210,212]. These polymers could therefore be used to design the proposed nanovaccine, taking advantage of the intrinsic adjuvanticity [213,214,215,216,217] and immunomodulating properties in terms of Th1/Th2 balance [214,215,216,217] of such systems—representing a clear advantage over the molecular adjuvants proposed thus far for gingipain-based vaccines [209,218]. Our research group has consistently demonstrated the immunogenic potential of polyester- and chitosan-based microparticles and nanoparticles for mucosal vaccination against various pathogens [210,213,214,215,216,217]. These studies highlight how encapsulating or adsorbing antigens onto polymeric systems can efficiently elicit robust antibody titers, local mucosal responses, and T-cell-mediated immunity, providing protection against experimental infections. Florindo et al. explored PLA nanospheres as carriers for *Streptococcus equi* antigens, showing significant induction of mixed Th1/Th2 responses crucial for protective immunity in both mucosal and systemic compartments [214]. The integration of mucoadhesive polymers like chitosan into PLA nanocarriers further optimised mucosal delivery and antigen uptake [214,216]. Similarly, the encapsulation of antigens in PLGA microspheres provided enhanced antigen stability and targeted delivery to APCs, eliciting robust immune responses [213]. Exploration of chitosan nanocarriers for tuberculosis also demonstrated enhanced interaction with macrophages and mucosal immune activation [217]. Although these studies targeted diseases other than AD (e.g., *Streptococcus equi* in equine strangles or *Mycobacterium bovis* in BCG vaccines), the underlying principles of mucosal immunisation and polymeric carrier systems are highly relevant to developing a nanovaccine for *Pg* as a prophylactic strategy against AD. In this context, our recent experimental study developed a chitosan-coated PLGA nanovaccine for mucosal delivery of *Pg* antigens, representing the first use of nanotechnology to address the link between PeD and AD. While in vivo validation was not performed, the nanovaccine demonstrated promising physicochemical, mucoadhesive, and antigen release properties for oral delivery, effective antigen protection, and successful in vitro uptake with minimal cytotoxicity, collectively underscoring the potential of these nanocarriers as vaccine candidates against *Pg* [219].

Overall, these findings suggest that incorporating *Pg*-specific antigens into oral polymeric nanocarriers could be a promising approach to elicit both local and systemic immunity, potentially blocking *Pg* dissemination and the associated inflammatory response implicated in AD. Harnessing the flexibility of nanotechnology may allow for the co-delivery of additional immunomodulators or adjuvants specifically tailored to AD pathology [212], further enhancing the prophylactic potential of the vaccine. Determining who should receive such a vaccine and when remains unclear, pending further epidemiological data and validated predictive biomarkers. Nonetheless, it is anticipated that the prophylactic vaccine would need to be administered during the early, reversible, preclinical stages of AD [128,204], possibly exploiting the 10-year timeline in AD risk development following PeD diagnosis. If successful, this approach could represent a significant paradigm shift, linking oral health interventions, immunoprophylaxis, and neurodegenerative disease prevention.

## 6. Conclusions

The present review highlights the growing evidence linking PeD to AD, suggesting that PeD may act as an upstream driver of AD. Among the variety of periodontal pathogens that may synergistically contribute to AD pathogenesis, *Pg* is hypothesised to play a keystone pathogenic role in the brain, similar to its established role in the subgingival biofilm, exerting damaging effects on the BBB and potentially contributing to the emergence and persistence of microbial communities within the brain. Equipped with a repertoire of virulence factors, this bacterium likely influences the pathophysiology of AD, activating specific mechanistic pathways to produce the full spectrum of responses characteristic of the disease, including pathways relevant for systemic and intracerebral production of Aβ upon infection. These findings support a microbial contribution to AD pathogenesis and align with the antimicrobial protection hypothesis, a revised infection hypothesis for AD, where Aβ release is seen as a protective mechanism against infection, eventually becoming pathogenic as it accumulates.

Understanding the interplay between PeD and AD highlights opportunities for intervention. Among potential strategies, the development of an oral nanoparticulate vaccine targeting *Pg* holds promise. By eliciting both mucosal and systemic immunity, such a vaccine could inhibit *Pg* progression to the brain, mitigating its role in AD. While future preclinical, epidemiological, and clinical research will be necessary to fully unravel the actual potential of this and other methods for reducing the risk of AD, we urge the inclusion of PeD in the life-course model of potentially modifiable risk factors for dementia prevention and call upon governments to recognise oral health as a major contributor to systemic health, making dental care more accessible to the general population.

## Data Availability

Not applicable.

## Abbreviations

ACh, acetylcholine; AChE, acetylcholinesterase; AChEIs, acetylcholinesterase inhibitors; AD, Alzheimer’s disease; ADRD, Alzheimer’s disease-related dementias; AMP, antimicrobial peptide; APCs, antigen-presenting cells; APP, amyloid β precursor protein; Aβ, amyloid beta; BACE1, beta-secretase 1; BBB, blood–brain barrier; CNS, central nervous system; CSF, cerebrospinal fluid; FAD, familial Alzheimer’s disease; GALT, gut-associated lymphoid tissue; GSK3β, glycogen synthase kinase 3 beta; GWAS, genome-wide association studies; HCV, hepatitis C virus; IL, interleukin; KEGG, Kyoto Encyclopedia of Genes and Genomes; Kgp, lysine-specific gingipain; LPS, lipopolysaccharide; MCI, mild cognitive impairment; MR, Mendelian randomisation; NFTs, neurofibrillary tangles; OMVs, outer membrane vesicles; PCL, poly-ε-caprolactone; PeD, periodontitis; *Pg*, *Porphyromonas gingivalis*; *Pg*-LPS, *Porphyromonas gingivalis* lipopolysaccharide; PLA, polylactic acid; PLGA, poly(lactic-co-glycolic acid); PPD, probing pocket depth; PSEN1, presenilin 1; PSEN2, presenilin 2; p-tau, phosphorylated tau; RgpA, arginine-specific gingipain A; RgpB, arginine-specific gingipain B; SAD, sporadic Alzheimer’s disease; TLR, Toll-like receptor; TNF-α, tumour necrosis factor alpha; t-tau, total tau.

## References

[1] Hippius H., Gabriele N. (2003). The discovery of Alzheimer’s Disease. Dialogues Clin. Neurosci..

[2] Federspiel C., Keipes M. (2014). Geriatrics from the 19th to the 21st century. 150 years of geriatric medicine: From increasing life expectancy to improving quality of life for the very old. Bull. Soc. Sci. Med. Grand. Duche. Luxemb..

[3] Alzheimer’s Association (2020). 2020 Alzheimer’s disease facts and figures. Alzheimer’s Dement..

[4] Lindemer E.R., Greve D.N., Fischl B.R., Augustinack J.C., Salat D.H. (2017). Regional staging of white matter signal abnormalities in aging and Alzheimer’s disease. Neuroimage Clin..

[5] Tom S.E., Hubbard R.A., Crane P.K., Haneuse S.J., Bowen J., McCormick W.C., McCurry S., Larson E.B. (2015). Characterization of dementia and Alzheimer’s disease in an older population: Updated incidence and life expectancy with and without dementia. Am. J. Public Health.

[6] Gauthier S., Webster C., Servaes S., Morais J.A., Rosa-Neto P. (2022). World Alzheimer Report 2022: Life after Diagnosis: Navigating Treatment, Care and Support.

[7] Patterson C. (2018). World Alzheimer Report 2018: The State of the Art of Dementia Research: New Frontiers.

[8] Baumgart M., Snyder H., Carrillo M., Fazio S., Kim H., Johns H. (2015). Summary of the evidence on modifiable risk factors for cognitive decline and dementia: A population-based perspective. Alzheimer’s Dement..

[9] Livingston G., Huntley J., Sommerlad A., Ames D., Ballard C., Banerjee S., Brayne C., Burns A., Cohen-Mansfield J., Cooper C. (2020). Dementia prevention, intervention, and care: 2020 Report of the Lancet Commission. Lancet.

[10] Livingston G., Huntley J., Liu K.Y., Costafreda S.G., Selbæk G., Alladi S., Ames D., Banerjee S., Burns A., Brayne C. (2024). Dementia prevention, intervention, and care: 2024 report of the Lancet standing Commission. Lancet.

[11] Dominy S.S., Lynch C., Ermini F., Benedyk M., Marczyk A., Konradi A., Nguyen M., Haditsch U., Raha D., Griffin C. (2019). *Porphyromonas gingivalis* in Alzheimer’s disease brains: Evidence for disease causation and treatment with small-molecule inhibitors. Sci. Adv..

[12] Carter C.J., France J., Crean S., Singhrao S.K. (2017). The *Porphyromonas gingivalis*/host interactome shows enrichment in GWASdb genes related to Alzheimer’s disease, diabetes and cardiovascular diseases. Front. Aging Neurosci..

[13] Chen C.-K., Wu Y.-T., Chang Y.-C. (2017). Association between chronic periodontitis and the risk of Alzheimer’s disease: A retrospective, population-based, matched-cohort study. Alzheimer’s Res. Ther..

[14] Panza F., Lozupone M., Solfrizzi V., Watling M., Imbimbo B.P. (2019). Time to test antibacterial therapy in Alzheimer’s disease. Brain.

[15] Soscia S.J., Kirby J.E., Washicosky K.J., Tucker S.M., Ingelsson M., Hyman B., Burton M.A., Goldstein L.E., Duong S., Tanzi R.E. (2010). The Alzheimer’s disease-associated amyloid beta-protein is an antimicrobial peptide. PLoS ONE.

[16] Moir R.D., Lathe R., Tanzi R.E. (2018). The antimicrobial protection hypothesis of Alzheimer’s disease. Alzheimer’s Dement..

[17] Eratne D., Loi S.M., Farrand S., Kelso W., Velakoulis D., Looi J.C.L. (2018). Alzheimer’s disease: Clinical update on epidemiology, pathophysiology and diagnosis. Australas. Psychiatry.

[18] Jack C.R., Albert M.S., Knopman D.S., McKhann G.M., Sperling R.A., Carrillo M.C., Thies B., Phelps C.H. (2011). Introduction to the recommendations from the National Institute on Aging-Alzheimer’s Association workgroups on diagnostic guidelines for Alzheimer’s disease. Alzheimer’s Dement..

[19] Bateman R., Aisen P., Strooper B., Fox N., Lemere C., Ringman J., Salloway S., Sperling R., Windisch M., Xiong C. (2011). Autosomal-dominant Alzheimer’s disease: A review and proposal for the prevention of Alzheimer’s disease. Alzheimer’s Res. Ther..

[20] Pritchard A.B., Crean S., Olsen I., Singhrao S.K. (2017). Periodontitis, microbiomes and their role in Alzheimer’s disease. Front. Aging Neurosci..

[21] Alzheimer’s Association (2021). 2021 Alzheimer’s disease facts and figures. Alzheimer’s Dement..

[22] Jack C.R., Knopman D.S., Jagust W.J., Petersen R.C., Weiner M.W., Aisen P.S., Shaw L.M., Vemuri P., Wiste H.J., Weigand S.D. (2013). Tracking pathophysiological processes in Alzheimer’s disease: An updated hypothetical model of dynamic biomarkers. Lancet Neurol..

[23] Davies P., Maloney A. (1976). Selective loss of central cholinergic neurons in Alzheimer’s disease. Lancet.

[24] Ferreira-Vieira T.H., Guimaraes I.M., Silva F.R., Ribeiro F.M. (2016). Alzheimer’s disease: Targeting the cholinergic system. Curr. Neuropharmacol..

[25] Liu P.P., Xie Y., Meng X.-Y., Kang J.-S. (2019). History and progress of hypotheses and clinical trials for Alzheimer’s disease. Signal Transduct. Target. Ther..

[26] Hardy J.A., Allsop D. (1991). Amyloid deposition as the central event in the aetiology of Alzheimer’s disease. Trends Pharmacol. Sci..

[27] Hardy J.A., Higgins G.A. (1992). Alzheimer’s disease: The amyloid cascade hypothesis. Science.

[28] Paula V.J.R., Guimarães F.M., Diniz B.S., Forlenza O.V. (2009). Neurobiological pathways to Alzheimer’s disease: Amyloid-beta, tau protein or both?. Dement. Neuropsychol..

[29] Fan L., Mao C., Hu X., Zhang S., Yang Z., Hu Z., Sun H., Fan Y., Dong Y., Yang J. (2020). New insights into the pathogenesis of Alzheimer’s disease. Front. Neurol..

[30] Selkoe D.J. (2008). Soluble oligomers of the amyloid β-protein impair synaptic plasticity and behavior. Behav. Brain Res..

[31] Ferreira S.T., Klein W.L. (2011). The Aβ oligomer hypothesis for synapse failure and memory loss in Alzheimer’s disease. Neurobiol. Learn. Mem..

[32] Zhang Y., Chen H., Li R., Sterling K., Song W. (2023). Amyloid β-based therapy for Alzheimer’s disease: Challenges, successes and future. Signal Transduct. Target. Ther..

[33] Busche M.A., Wegmann S., Dujardin S., Commins C., Schiantarelli J., Klickstein N., Kamath T.V., Carlson G.A., Nelken I., Hyman B.T. (2019). Tau impairs neural circuits, dominating amyloid-β effects, in Alzheimer models in vivo. Nat. Neurosci..

[34] Swerdlow R.H., Khan S.M. (2004). A “mitochondrial cascade hypothesis” for sporadic Alzheimer’s disease. Med. Hypotheses.

[35] Scheffer S., Hermkens D.M.A., van der Weerd L., De Vries H.E., Daemen M.J.A.P. (2021). Vascular hypothesis of Alzheimer disease. Arterioscler. Thromb. Vasc. Biol..

[36] Chen L.-L., Fan Y.-G., Zhao L.-X., Zhang Q., Wang Z.-Y. (2023). The metal ion hypothesis of Alzheimer’s disease and the anti-neuroinflammatory effect of metal chelators. Bioorg. Chem..

[37] Praticò D. (2008). Oxidative stress hypothesis in Alzheimer’s disease: A reappraisal. Trends Pharmacol. Sci..

[38] Morgen K., Frölich L. (2015). The metabolism hypothesis of Alzheimer’s disease: From the concept of central insulin resistance and associated consequences to insulin therapy. J. Neural. Transm..

[39] Hooper C., Killick R., Lovestone S. (2008). The GSK3β hypothesis of Alzheimer’s disease. J. Neurochem..

[40] Cribbs D.H., Berchtold N.C., Perreau V., Coleman P.D., Rogers J., Tenner A.J., Cotman C.W. (2012). Extensive innate immune gene activation accompanies brain aging, increasing vulnerability to cognitive decline and neurodegeneration: A microarray study. J. Neuroinflamm..

[41] Zhang B., Gaiteri C., Bodea L.-G., Wang Z., McElwee J., Podtelezhnikov A.A., Zhang C., Xie T., Tran L., Dobrin R. (2013). Integrated systems approach identifies genetic nodes and networks in late-onset Alzheimer’s disease. Cell.

[42] Song W., Hooli B., Mullin K., Jin S.C., Cella M., Ulland T.K., Wang Y., Tanzi R.E., Colonna M. (2017). Alzheimer’s disease-associated TREM2 variants exhibit either decreased or increased ligand-dependent activation. Alzheimer’s Dement..

[43] Du X., Wang X., Geng M. (2018). Alzheimer’s disease hypothesis and related therapies. Transl. Neurodegener..

[44] Carter C.J. (2017). Genetic, transcriptome, proteomic, and epidemiological evidence for blood-brain barrier disruption and polymicrobial brain invasion as determinant factors in Alzheimer’s disease. J. Alzheimer’s Dis. Rep..

[45] Miklossy J. (2011). Alzheimer’s disease—A neurospirochetosis. Analysis of the evidence following Koch’s and Hill’s criteria. J. Neuroinflamm..

[46] Pisa D., Alonso R., Rábano A., Horst M.N., Carrasco L. (2016). Fungal enolase, β-tubulin, and chitin are detected in brain tissue from Alzheimer’s disease patients. Front. Microbiol..

[47] Seaks C.E., Wilcock D.M. (2020). Infectious hypothesis of Alzheimer disease. PLoS Pathog..

[48] Kumar D.K.V., Choi S.H., Washicosky K.J., Eimer W.A., Tucker S., Ghofrani J., Lefkowitz A., McColl G., Goldstein L.E., Tanzi R.E. (2016). Amyloid-β peptide protects against microbial infection in mouse and worm models of Alzheimer’s disease. Sci. Transl. Med..

[49] White M.R., Kandel R., Tripathi S., Condon D., Qi L., Taubenberger J., Hartshorn K.L. (2014). Alzheimer’s associated β-amyloid protein inhibits influenza A virus and modulates viral interactions with phagocytes. PLoS ONE.

[50] Bourgade K., Le Page A., Bocti C., Witkowski J.M., Dupuis G., Frost E.H., Fülöp T. (2016). Protective effect of amyloid-β peptides against herpes simplex virus-1 infection in a neuronal cell culture model. J. Alzheimer’s Dis..

[51] Jaeger L.B., Dohgu S., Sultana R., Lynch J.L., Owen J.B., Erickson M.A., Shah G.N., Price T.O., Fleegal-Demotta M.A., Butterfiled D.A. (2009). Lipopolysaccharide alters the blood-brain barrier transport of amyloid β protein: A mechanism for inflammation in the progression of Alzheimer’s disease. Brain Behav. Immun..

[52] Frost G.R., Li Y.-M. (2017). The role of astrocytes in amyloid production and Alzheimer’s disease. Open Biol..

[53] Fulop T., Witkowski J.M., Bourgade K., Khalil A., Zerif E., Larbi A., Hirokawa K., Pawelec G., Bocti C., Lacombe G. (2018). Can an infection hypothesis explain the beta amyloid hypothesis of Alzheimer’s disease?. Front. Aging Neurosci..

[54] Itzhaki R.F. (2016). Herpes and Alzheimer’s disease: Subversion in the central nervous system and how it might be halted. J. Alzheimer’s Dis..

[55] Zhan X., Stamova B., Jin L.-W., DeCarli C., Phinney B., Sharp F.R. (2016). Gram-negative bacterial molecules associate with Alzheimer disease pathology. Neurology.

[56] Emery D.C., Shoemark D.K., Batstone T.E., Waterfall C.M., Coghill J.A., Cerajewska T.L., Davies M., West N.X., Allen S.J. (2017). 16S rRNA next generation sequencing analysis shows bacteria in Alzheimer’s post-mortem brain. Front. Aging Neurosci..

[57] Alonso R., Pisa D., Aguado B., Carrasco L. (2017). Identification of fungal species in brain tissue from Alzheimer’s disease by next-generation sequencing. J. Alzheimer’s Dis..

[58] Wu S.C., Cao Z.-S., Chang K.-M., Juang J.-L. (2017). Intestinal microbial dysbiosis aggravates the progression of Alzheimer’s disease in Drosophila. Nat. Commun..

[59] Liu X.X., Jiao B., Liao X.-X., Guo L.-N., Yuan Z.-H., Wang X., Xiao X.-W., Zhang X.-Y., Tang B.-S., Shen L. (2019). Analysis of salivary microbiome in patients with Alzheimer’s disease. J. Alzheimer’s Dis..

[60] Singhrao S.K., Olsen I. (2019). Assessing the role of *Porphyromonas gingivalis* in periodontitis to determine a causative relationship with Alzheimer’s disease. J. Oral Microbiol..

[61] Hajishengallis G., Chavakis T. (2021). Local and systemic mechanisms linking periodontal disease and inflammatory comorbidities. Nat. Rev. Immunol..

[62] Vos T., Abajobir A.A., Abate K.H., Abbafati C., Abbas K.M., Abd-Allah F., Abdulkader R.S., Abdulle A.M., Abebo T.A., Abera S.F. (2017). Global, regional, and national incidence, prevalence, and years lived with disability for 328 diseases and injuries for 195 countries, 1990–2016: A systematic analysis for the Global Burden of Disease Study 2016. Lancet.

[63] Nazir M., Al-Ansari A., Al-Khalifa K., Alhareky M., Gaffar B., Almas K. (2020). Global prevalence of periodontal disease and lack of its surveillance. Sci. World J..

[64] Papapanou P.N. (2012). The prevalence of periodontitis in the US. J. Dent. Res..

[65] Hajishengallis G. (2014). Immunomicrobial pathogenesis of periodontitis: Keystones, pathobionts, and host response. Trends Immunol..

[66] Moutsopoulos N.M., Konkel J.E. (2018). Tissue-specific immunity at the oral mucosal barrier. Trends Immunol..

[67] Hajishengallis G., Lamont R.J. (2012). Beyond the red complex and into more complexity: The polymicrobial synergy and dysbiosis (PSD) model of periodontal disease etiology. Mol. Oral Microbiol..

[68] Arora N., Mishra A., Chugh S. (2014). Microbial role in periodontitis: Have we reached the top? Some unsung bacteria other than red complex. J. Indian Soc. Periodontol..

[69] Holt S.C., Ebersole J., Felton J., Brunsvold M., Kornman K.S. (1988). Implantation of *Bacteroides gingivalis* in nonhuman primates initiates progression of periodontitis. Science.

[70] Miller D.P., Hutcherson J.A., Wang Y., Nowakowska Z.M., Potempa J., Yoder-Himes D.R., Scott D.A., Whiteley M., Lamont R.J. (2017). Genes contributing to *Porphyromonas gingivalis* fitness in abscess and epithelial cell colonization environments. Front. Cell. Infect. Microbiol..

[71] Hajishengallis G., Lamont R.J. (2014). Breaking bad: Manipulation of the host response by *Porphyromonas gingivalis*. Eur. J. Immunol..

[72] Holt S.C., Kesavalu L., Walker S., Genco C.A. (1999). Virulence factors of *Porphyromonas gingivalis*. Periodontol. 2000.

[73] Jia L., Han N., Du J., Guo L., Luo Z., Liu Y. (2019). Pathogenesis of important virulence factors of *Porphyromonas gingivalis* via toll-like receptors. Front. Cell. Infect. Microbiol..

[74] Singh A., Wyant T., Anaya-Bergman C., Aduse-Opoku J., Brunner J., Laine M.L., Curtis M.A., Lewis J.P. (2011). The capsule of *Porphyromonas gingivalis* leads to a reduction in the host inflammatory response, evasion of phagocytosis, and increase in virulence. Infect. Immun..

[75] Enersen M., Nakano K., Amano A. (2013). *Porphyromonas gingivalis* fimbriae. J. Oral Microbiol..

[76] Zhang Z., Liu D., Liu S., Zhang S., Pan Y. (2021). The role of *Porphyromonas gingivalis* outer membrane vesicles in periodontal disease and related systemic diseases. Front. Cell. Infect. Microbiol..

[77] Nakayama M., Ohara N. (2017). Molecular mechanisms of *Porphyromonas gingivalis*-host cell interaction on periodontal diseases. Jpn. Dent. Sci. Rev..

[78] Riviere G.R., Riviere K.H., Smith K.S. (2002). Molecular and immunological evidence of oral *Treponema* in the human brain and their association with Alzheimer’s disease. Oral Microbiol. Immunol..

[79] Noble J.M., Scarmeas N., Celenti R.S., Elkind M.S.V., Wright C.B., Schupf N., Papapanou P.N. (2014). Serum IgG antibody levels to periodontal microbiota are associated with incident Alzheimer disease. PLoS ONE.

[80] Kondo K., Niino M., Shido K. (1994). A case-control study of Alzheimer’s disease in Japan—Significance of life-styles. Dement. Geriatr. Cogn. Disord..

[81] Ship J.A., Puckett S.A. (1994). Longitudinal study on oral health in subjects with Alzheimer’s disease. J. Am. Geriatr. Soc..

[82] Ma K.S., Hasturk H., Carreras I., Dedeoglu A., Veeravalli J.J., Huang J.Y., Kantarci A., Wei J.C. (2022). Dementia and the risk of periodontitis: A population-based cohort study. J. Dent. Res..

[83] Gatz M., Mortimer J.A., Fratiglioni L., Johansson B., Berg S., Reynolds C.A., Pedersen N.L. (2006). Potentially modifiable risk factors for dementia in identical twins. Alzheimer’s Dement..

[84] Sparks Stein P., Desrosiers M., Donegan S.J., Yepes J.F., Kryscio R.J. (2007). Tooth loss, dementia and neuropathology in the Nun Study. J. Am. Dent. Assoc..

[85] Popovac A., Čelebić A., Peršić S., Stefanova E., Milić Lemić A., Stančić I. (2021). Oral health status and nutritional habits as predictors for developing Alzheimer’s disease. Med. Princ. Pract..

[86] Alfotawi R., Alzahrani S., Alhefdhi R., Altamimi A., Alfadhel A., Alshareef A., Aldawsari B., Sonbol S., Alsubaie F., Alwahibi A. (2020). The relation between teeth loss and cognitive decline among Saudi population in the city of Riyadh: A pilot study. Saudi Dent. J..

[87] Asher S., Stephen R., Ngandu T., Koskinen S., Suominen A.L., Solomon A. (2021). Association between tooth loss and cognitive performance: 11-year follow-up cohort study. Alzheimer’s Dement..

[88] Chava V.K., Nuvvula S., Nuvvula S. (2016). Primary culprit for tooth loss!!. J. Indian Soc. Periodontol..

[89] Kim Y.-T., Choi J.-K., Kim D.-H., Jeong S.-N., Lee J.-H. (2019). Association between health status and tooth loss in Korean adults: Longitudinal results from the National Health Insurance Service-Health Examinee Cohort, 2002–2015. J. Periodontal Implant Sci..

[90] Sun Y.-Q., Richmond R.C., Chen Y., Mai X.-M. (2020). Mixed evidence for the relationship between periodontitis and Alzheimer’s disease: A bidirectional Mendelian randomization study. PLoS ONE.

[91] Jiang Z., Shi Y., Zhao W., Zhou L., Zhang B., Xie Y., Zhang Y., Tan G., Wang Z. (2021). Association between chronic periodontitis and the risk of Alzheimer’s disease: Combination of text mining and GEO dataset. BMC Oral Health.

[92] Jin J., Guang M., Ogbuehi A.C., Li S., Zhang K., Ma Y., Acharya A., Guo B., Peng Z., Liu X. (2021). Shared molecular mechanisms between Alzheimer’s disease and periodontitis revealed by transcriptomic analysis. Biomed. Res. Int..

[93] Kaye E.K., Valencia A., Baba N., Spiro A., Dietrich T., Garcia R.I. (2010). Tooth loss and periodontal disease predict poor cognitive function in older men. J. Am. Geriatr. Soc..

[94] Iwasaki M., Yoshihara A., Kimura Y., Sato M., Wada T., Sakamoto R., Ishimoto Y., Fukutomi E., Chen W., Imai H. (2016). Longitudinal relationship of severe periodontitis with cognitive decline in older Japanese. J. Periodontal Res..

[95] Iwasaki M., Kimura Y., Ogawa H., Yamaga T., Ansai T., Wada T., Sakamoto R., Ishimoto Y., Fujisawa M., Okumiya K. (2019). Periodontitis, periodontal inflammation, and mild cognitive impairment: A 5-year cohort study. J. Periodontal Res..

[96] Tzeng N.-S., Chung C.-H., Yeh C.-B., Huang R.-Y., Yuh D.-Y., Huang S.-Y., Lu R.-B., Chang H.-A., Kao Y.-C., Chiang W.-S. (2016). Are chronic periodontitis and gingivitis associated with dementia? A nationwide, retrospective, matched-cohort study in Taiwan. Neuroepidemiology.

[97] Lee Y.T., Lee H.-C., Hu C.-J., Huang L.-K., Chao S.-P., Lin C.-P., Su E.C.-Y., Lee Y.-C., Chen C.-C. (2017). Periodontitis as a modifiable risk factor for dementia: A nationwide population-based cohort study. J. Am. Geriatr. Soc..

[98] Lee Y.L., Hu H.-Y., Huang L.-Y., Chou P., Chu D. (2017). Periodontal disease associated with higher risk of dementia: Population-based cohort study in Taiwan. J. Am. Geriatr. Soc..

[99] De Souza Rolim T., Fabri G.M.C., Nitrini R., Anghinah R., Teixeira M.J., De Siqueira J.T.T., Cestari J.A.F., De Siqueira S.R.D.T. (2014). Evaluation of patients with Alzheimer’s disease before and after dental treatment. Arq. Neuropsiquiatr..

[100] Choi S., Kim K., Chang J., Kim S.M., Kim S.J., Cho H., Park S.M. (2019). Association of chronic periodontitis on Alzheimer’s disease or vascular dementia. J. Am. Geriatr. Soc..

[101] Chiu W.-C., Tsan Y.-T., Tsai S.-L., Chang C.-J., Wang J.-D., Chen P.-C. (2014). Hepatitis C viral infection and the risk of dementia. Eur. J. Neurol..

[102] Malone J., Jung J., Tran L., Zhao C. (2022). Periodontal disease and risk of dementia in Medicare patients with hepatitis C virus. J. Alzheimer’s Dis..

[103] Nikitin B., Bahorik A.L., Mcevoy C.T., Yaffe K. (2021). Markers of periodontal disease and cognitive decline in a diverse, older cohort. Alzheimer’s Dement..

[104] Nilsson H., Berglund J.S., Renvert S. (2018). Periodontitis, tooth loss and cognitive functions among older adults. Clin. Oral Investig..

[105] Nilsson H., Berglund J.S., Renvert S. (2018). Longitudinal evaluation of periodontitis and development of cognitive decline among older adults. J. Clin. Periodontol..

[106] Shin H.-S., Shin M.-S., Ahn Y.-B., Choi B.-Y., Nam J.-H., Kim H.-D. (2016). Periodontitis is associated with cognitive impairment in elderly Koreans: Results from the Yangpyeong cohort study. J. Am. Geriatr. Soc..

[107] Kamer A.R., Morse D.E., Holm-Pedersen P., Mortensen E.L., Avlund K. (2012). Periodontal inflammation in relation to cognitive function in an older adult Danish population. J. Alzheimer’s Dis..

[108] Zhang S., Yang F., Wang Z., Qian X., Ji Y., Gong L., Ge S., Yan F. (2020). Poor oral health conditions and cognitive decline: Studies in humans and rats. PLoS ONE.

[109] Marruganti C., Baima G., Aimetti M., Grandini S., Sanz M., Romandini M. (2023). Periodontitis and low cognitive performance: A population-based study. J. Clin. Periodontol..

[110] Gil-Montoya J.A., Sanchez-Lara I., Carnero-Pardo C., Fornieles F., Montes J., Vilchez R., Burgos J.S., González-Moles M.A., Barrios R., Bravo M. (2015). Is periodontitis a risk factor for cognitive impairment and dementia? A case-control study. J. Periodontol..

[111] Gil-Montoya J.A., Barrios R., Santana S., Sanchez-Lara I., Carnero-Pardo C., Francisco F., Montes J., Ramírez C., González-Moles M.A., Burgos J.S. (2017). Association between periodontitis and amyloid β peptide in elderly people with and without cognitive impairment. J. Periodontol..

[112] Holmer J., Eriksdotter M., Schultzberg M., Pussinen P.J., Buhlin K. (2018). Association between periodontitis and risk of Alzheimer’s disease, mild cognitive impairment and subjective cognitive decline: A case-control study. J. Clin. Periodontol..

[113] De Souza Rolim T., Fabri G.M.C., Nitrini R., Anghinah R., Teixeira M.J., De Siqueira J.T.T., Cestari J.A.F., De Siqueira S.R.D.T. (2014). Oral infections and orofacial pain in Alzheimer’s disease: A case-control study. J. Alzheimer’s Dis..

[114] De Oliveira Araújo R., Villoria G.E.M., Luiz R.R., Esteves J.C., Leão A.T.T., Feres-Filho E.J. (2021). Association between periodontitis and Alzheimer’s disease and its impact on the self-perceived oral health status: A case-control study. Clin. Oral Investig..

[115] Panzarella V., Mauceri R., Baschi R., Maniscalco L., Campisi G., Monastero R. (2022). Oral health status in subjects with amnestic mild cognitive impairment and Alzheimer’s disease: Data from the Zabút Aging Project. J. Alzheimer’s Dis..

[116] Tiisanoja A., Syrjälä A.-M., Tertsonen M., Komulainen K., Pesonen P., Knuuttila M., Hartikainen S., Ylöstalo P. (2019). Oral diseases and inflammatory burden and Alzheimer’s disease among subjects aged 75 years or older. Spec. Care Dentist..

[117] Stewart R., Weyant R.J., Garcia M.E., Harris T., Launer L.J., Satterfield S., Simonsick E.M., Yaffe K., Newman A.B. (2013). Adverse oral health and cognitive decline: The health, aging and body composition study. J. Am. Geriatr. Soc..

[118] Holmer J., Eriksdotter M., Häbel H., Hed Myrberg I., Jonsson A., Pussinen P.J., Garcia-Ptacek S., Jansson L., Sandborgh-Englund G., Buhlin K. (2022). Periodontal conditions and incident dementia: A nationwide Swedish cohort study. J. Periodontol..

[119] Arrivé E., Letenneur L., Matharan F., Laporte C., Helmer C., Barberger-Gateau P., Miquel J.L., Dartigues J.F. (2012). Oral health condition of French elderly and risk of dementia: A longitudinal cohort study. Community Dent. Oral Epidemiol..

[120] Asher S., Stephen R., Suominen A.L., Mäntylä P., Solomon A. (2020). Association of periodontitis and cognitive impairment: A systematic review and meta-analysis. Alzheimer’s Dement..

[121] Torrealba-García D., Garcia-Morales P., Torrealba E., Cejudo J.C., Silvestre-Rangil J. (2021). Is there a relationship between periodontitis and Alzheimer’s disease? Systematic review and comparative analysis. Alzheimer’s Dement..

[122] Hu X., Zhang J., Qiu Y., Liu Z. (2021). Periodontal disease and the risk of Alzheimer’s disease and mild cognitive impairment: A systematic review and meta-analysis. Psychogeriatrics.

[123] Sparks Stein P., Steffen M.J., Smith C., Jicha G., Ebersole J.L., Abner E., Dawson D. (2012). Serum antibodies to periodontal pathogens are a risk factor for Alzheimer’s disease. Alzheimer’s Dement..

[124] Davies N.M., Holmes M.V., Davey Smith G. (2018). Reading Mendelian randomisation studies: A guide, glossary, and checklist for clinicians. BMJ.

[125] Kim D.-H., Jeong S.-N., Lee J.-H. (2020). Severe periodontitis with tooth loss as a modifiable risk factor for the development of Alzheimer, vascular, and mixed dementia: National Health Insurance Service-National Health Screening Retrospective Cohort 2002–2015. J. Periodontal Implant Sci..

[126] Demmer R.T., Norby F.L., Lakshminarayan K., Walker K.A., Pankow J.S., Folsom A.R., Mosley T., Beck J., Lutsey P.L. (2020). Periodontal disease and incident dementia: The Atherosclerosis Risk in Communities Study (ARIC). Neurology.

[127] Beydoun M.A., Beydoun H.A., Hossain S., El-Hajj Z.W., Weiss J., Zonderman A.B. (2020). Clinical and bacterial markers of periodontitis and their association with incident all-cause and Alzheimer’s disease dementia in a large national survey. J. Alzheimer’s Dis..

[128] Kamer A.R., Craig R.G., Niederman R., Fortea J., De Leon M.J. (2020). Periodontal disease as a possible cause for Alzheimer’s disease. Periodontol. 2000.

[129] Kamer A.R., Craig R.G., Pirraglia E., Dasanayake A.P., Norman R.G., Boylan R.J., Nehorayoff A., Glodzik L., Brys M., De Leon M.J. (2009). TNF-α and antibodies to periodontal bacteria discriminate between Alzheimer’s disease patients and normal subjects. J. Neuroimmunol..

[130] Farhad S.Z., Amini S., Khalilian A., Barekatain M., Mafi M., Barekatain M., Rafei E. (2014). The effect of chronic periodontitis on serum levels of tumor necrosis factor-alpha in Alzheimer disease. Dent. Res. J. (Isfahan).

[131] Cestari J.A.F., Fabri G.M.C., Kalil J., Nitrini R., Jacob-Filho W., De Siqueira J.T.T., De Siqueira S.R.D.T. (2016). Oral infections and cytokine levels in patients with Alzheimer’s disease and mild cognitive impairment compared with controls. J. Alzheimer’s Dis..

[132] Ide M., Harris M., Stevens A., Sussams R., Hopkins V., Culliford D., Fuller J., Ibbett P., Raybould R., Thomas R. (2016). Periodontitis and cognitive decline in Alzheimer’s disease. PLoS ONE.

[133] Dye B.A., Herrera-Abreu M., Lerche-Sehm J., Vlachojannis C., Pikdoken L., Pretzl B., Schwartz A., Papapanou P.N. (2009). Serum antibodies to periodontal bacteria as diagnostic markers of periodontitis. J. Periodontol..

[134] Noble J.M., Borrell L.N., Papapanou P.N., Elkind M.S.V., Scarmeas N., Wright C.B. (2009). Periodontitis is associated with cognitive impairment among older adults: Analysis of NHANES-III. J. Neurol. Neurosurg. Psychiatry.

[135] Merchant A.T., Zhao L., Bawa E.M., Yi F., Vidanapathirana N.P., Lohman M., Zhang J. (2024). Association between clusters of antibodies against periodontal microorganisms and Alzheimer disease mortality: Evidence from a nationally representative survey in the USA. J. Periodontol..

[136] Laugisch O., Johnen A., Maldonado A., Ehmke B., Bürgin W., Olsen I., Potempa J., Sculean A., Duning T., Eick S. (2018). Periodontal pathogens and associated intrathecal antibodies in early stages of Alzheimer’s disease. J. Alzheimer’s Dis..

[137] Kamer A.R., Pirraglia E., Tsui W., Rusinek H., Vallabhajosula S., Mosconi L., Yi L., McHugh P., Craig R.G., Svetcov S. (2015). Periodontal disease associates with higher brain amyloid load in normal elderly. Neurobiol. Aging.

[138] Kamer A.R., Pushalkar S., Gulivindala D., Butler T., Li Y., Annam K.R.C., Glodzik L., Ballman K.V., Corby P.M., Blennow K. (2021). Periodontal dysbiosis associates with reduced CSF Aβ42 in cognitively normal elderly. Alzheimer’s Dement..

[139] Shoji M. (2019). Cerebrospinal fluid and plasma tau as a biomarker for brain tauopathy. Adv. Exp. Med. Biol..

[140] Carballo Á., López-Dequidt I., Custodia A., Botelho J., Aramburu-Núñez M., Machado V., Pías-Peleteiro J.M., Ouro A., Romaus-Sanjurjo D., Vázquez-Vázquez L. (2023). Association of periodontitis with cognitive decline and its progression: Contribution of blood-based biomarkers of Alzheimer’s disease to this relationship. J. Clin. Periodontol..

[141] Kubota T., Maruyama S., Abe D., Tomita T., Morozumi T., Nakasone N., Saku T., Yoshie H. (2014). Amyloid beta (A4) precursor protein expression in human periodontitis-affected gingival tissues. Arch. Oral Biol..

[142] Vlassenko A.G., McCue L., Jasielec M.S., Su Y., Gordon B.A., Xiong C., Holtzman D.M., Benzinger T.L.S., Morris J.C., Fagan A.M. (2016). Imaging and cerebrospinal fluid biomarkers in early preclinical Alzheimer disease. Ann. Neurol..

[143] Frenzel S., Wittfeld K., Habes M., Klinger-König J., Bülow R., Völzke H., Grabe H.J. (2020). A biomarker for Alzheimer’s disease based on patterns of regional brain atrophy. Front. Psychiatry.

[144] Franke K., Gaser C. (2019). Ten years of BrainAGE as a neuroimaging biomarker of brain aging: What insights have we gained?. Front. Neurol..

[145] Schwahn C., Frenzel S., Holtfreter B., van der Auwera S., Pink C., Bülow R., Friedrich N., Völzke H., Biffar R., Kocher T. (2022). Effect of periodontal treatment on preclinical Alzheimer’s disease—Results of a trial emulation approach. Alzheimer’s Dement..

[146] Adam H.S., Lakshminarayan K., Wang W., Norby F.L., Mosley T., Walker K.A., Gottesman R.F., Meyer K., Hughes T.M., Pankow J.S. (2022). The prospective association between periodontal disease and brain imaging outcomes: The Atherosclerosis Risk in Communities Study. J. Clin. Periodontol..

[147] Leblhuber F., Huemer J., Steiner K., Gostner J.M., Fuchs D. (2020). Knock-on effect of periodontitis to the pathogenesis of Alzheimer’s disease?. Wien. Klin. Wochenschr..

[148] Bathini P., Foucras S., Dupanloup I., Imeri H., Perna A., Berruex J., Doucey M., Annoni J., Auber Alberi L. (2020). Classifying dementia progression using microbial profiling of saliva. Alzheimer’s Dement..

[149] Wu Y.-F., Lee W.-F., Salamanca E., Yao W.-L., Su J.-N., Wang S.-Y., Hu C.-J., Chang W.-J. (2021). Oral microbiota changes in elderly patients, an indicator of Alzheimer’s disease. Int. J. Environ. Res. Public Health.

[150] Poole S., Singhrao S.K., Kesavalu L., Curtis M.A., Crean S. (2013). Determining the presence of periodontopathic virulence factors in short-term postmortem Alzheimer’s disease brain tissue. J. Alzheimer’s Dis..

[151] Bennett J.P., Keeney P.M., Brohawn D.G. (2019). RNA sequencing reveals small and variable contributions of infectious agents to transcriptomes of postmortem nervous tissues from amyotrophic lateral sclerosis, Alzheimer’s disease and Parkinson’s disease subjects, and increased expression of genes from disease-activated microglia. Front. Neurosci..

[152] Singhrao S.K., Harding A., Poole S., Kesavalu L., Crean S. (2015). *Porphyromonas gingivalis* periodontal infection and its putative links with Alzheimer’s disease. Mediat. Inflamm..

[153] van de Haar H.J., Burgmans S., Jansen J.F.A., van Osch M.J.P., van Buchem M.A., Muller M., Hofman P.A.M., Verhey F.R.J., Backes W.H. (2016). Blood-brain barrier leakage in patients with early Alzheimer disease. Radiology.

[154] Allen H.B., Morales D., Jones K., Joshi S. (2016). Alzheimer’s disease: A novel hypothesis integrating spirochetes, biofilm, and the immune system. J. Neuroinfect. Dis..

[155] Allen H.B. (2016). Alzheimer’s disease: Assessing the role of spirochetes, biofilms, the immune system, and amyloid-β with regard to potential treatment and prevention. J. Alzheimer’s Dis..

[156] Miklossy J. (2016). Bacterial amyloid and DNA are important constituents of senile plaques: Further evidence of the spirochetal and biofilm nature of senile plaques. J. Alzheimer’s Dis..

[157] Allen H.B., Morales D., Jones K., Joshi S. (2016). Alzheimer’s disease: A novel hypothesis for the development and the subsequent role of beta amyloid. J. Neuroinfect. Dis..

[158] Senejani A.G., Maghsoudlou J., El-Zohiry D., Gaur G., Wawrzeniak K., Caravaglia C., Khatri V.A., MacDonald A., Sapi E. (2022). *Borrelia burgdorferi* co-localizing with amyloid markers in Alzheimer’s disease brain tissues. J. Alzheimer’s Dis..

[159] Ng H.M., Slakeski N., Butler C.A., Veith P.D., Chen Y.-Y., Liu S.W., Hoffmann B., Dashper S.G., Reynolds E.C. (2019). The role of *Treponema denticola* motility in synergistic biofilm formation with *Porphyromonas gingivalis*. Front. Cell. Infect. Microbiol..

[160] Wozniak M., Mee A., Itzhaki R. (2009). Herpes simplex virus type 1 DNA is located within Alzheimer’s disease amyloid plaques. J. Pathol..

[161] Chen C., Feng P., Slots J. (2020). Herpesvirus-bacteria synergistic interaction in periodontitis. Periodontol. 2000.

[162] Guo Y., Nguyen K.-A., Potempa J. (2010). Dichotomy of gingipains action as virulence factors: From cleaving substrates with the precision of a surgeon’s knife to a meat chopper-like brutal degradation of proteins. Periodontol. 2000.

[163] Olsen I., Singhrao S.K. (2019). Poor oral health and its neurological consequences: Mechanisms of *Porphyromonas gingivalis* involvement in cognitive dysfunction. Curr. Oral Health Rep..

[164] Lobmaier I.V.K., Vege Å., Gaustad P., Rognum T.O. (2009). Bacteriological investigation—Significance of time lapse after death. Eur. J. Clin. Microbiol. Infect. Dis..

[165] Pfeffer R.I., Kurosaki T.T., Harrah C.H., Chance J.M., Filos S. (1982). Measurement of functional activities in older adults in the community. J. Gerontol..

[166] Petersen R.C., Stevens J.C., Ganguli M., Tangalos E.G., Cummings J.L., DeKosky S.T. (2001). Practice parameter: Early detection of dementia: Mild cognitive impairment (an evidence-based review). Neurology.

[167] Winblad B., Palmer K., Kivipelto M., Jelic V., Fratiglioni L., Wahlund L.-O., Nordberg A., Backman L., Albert M., Almkvist O. (2004). Mild cognitive impairment—Beyond controversies, towards a consensus: Report of the international working group on mild cognitive impairment. J. Intern. Med..

[168] Robles-Bayón A., Del Ser T., Alom J., Peña-Casanova J. (2002). Proposal of criteria for clinical diagnosis of mild cognitive impairment, dementia and Alzheimer’s disease. Neurologia.

[169] McKeith I.G., Galasko D., Kosaka K., Perry E.K., Dickson D.W., Hansen L.A., Salmon D.P., Lowe J., Mirra S.S., Byrne E.J. (1996). Consensus guidelines for the clinical and pathologic diagnosis of dementia with Lewy bodies (DLB). Neurology.

[170] Abbayya K., Puthanakar N.Y., Naduwinmani S., Chidambar Y.S. (2015). Association between periodontitis and Alzheimer’s disease. N. Am. J. Med. Sci..

[171] Wang R.P.-H., Huang J., Chan K.W.Y., Leung W.K., Goto T., Ho J.Y.S., Chang R.C.-C. (2023). IL-1β and TNF-α play an important role in modulating the risk of periodontitis and Alzheimer’s disease. J. Neuroinflamm..

[172] Liu Y., Wu Z., Zhang X., Ni J., Yu W., Zhou Y., Nakanishi H. (2013). Leptomeningeal cells transduce peripheral macrophages inflammatory signal to microglia in response to *Porphyromonas gingivalis* LPS. Mediat. Inflamm..

[173] Sato N., Matsumoto T., Kawaguchi S., Seya K., Matsumiya T., Ding J., Aizawa T., Imaizumi T. (2022). *Porphyromonas gingivalis* lipopolysaccharide induces interleukin-6 and c-c motif chemokine ligand 2 expression in cultured hCMEC/D3 human brain microvascular endothelial cells. Gerodontology.

[174] Ma X., Shin Y.-J., Yoo J.-W., Park H.-S., Kim D.-H. (2023). Extracellular vesicles derived from *Porphyromonas gingivalis* induce trigeminal nerve-mediated cognitive impairment. J. Adv. Res..

[175] Olsen I., Singhrao S.K. (2015). Can oral infection be a risk factor for Alzheimer’s disease?. J. Oral Microbiol..

[176] Dando S.J., Mackay-Sim A., Norton R., Currie B.J., St. John J.A., Ekberg J.A.K., Batzloff M., Ulett G.C., Beacham I.R. (2014). Pathogens penetrating the central nervous system: Infection pathways and the cellular and molecular mechanisms of invasion. Clin. Microbiol. Rev..

[177] Olsen I. (2021). Possible effects of *Porphyromonas gingivalis* on the blood-brain barrier in Alzheimer’s disease. Expert Rev. Anti Infect. Ther..

[178] Singhrao S.K., Olsen I. (2018). Are *Porphyromonas gingivalis* outer membrane vesicles microbullets for sporadic Alzheimer’s disease manifestation?. J. Alzheimer’s Dis. Rep..

[179] Nara P.L., Sindelar D., Penn M.S., Potempa J., Griffin W.S.T. (2021). *Porphyromonas gingivalis* outer membrane vesicles as the major driver of and explanation for neuropathogenesis, the cholinergic hypothesis, iron dyshomeostasis, and salivary lactoferrin in Alzheimer’s disease. J. Alzheimer’s Dis..

[180] Yoshida K., Yoshida K., Seyama M., Hiroshima Y., Mekata M., Fujiwara N., Kudo Y., Ozaki K. (2023). *Porphyromonas gingivalis* outer membrane vesicles in cerebral ventricles activate microglia in mice. Oral Dis..

[181] Hao X., Li Z., Li W., Katz J., Michalek S.M., Barnum S.R., Pozzo-Miller L., Saito T., Saido T.C., Wang Q. (2022). Periodontal infection aggravates C1q-mediated microglial activation and synapse pruning in Alzheimer’s mice. Front. Immunol..

[182] Wu Z., Ni J., Liu Y., Teeling J.L., Takayama F., Collcutt A., Ibbett P., Nakanishi H. (2017). Cathepsin B plays a critical role in inducing Alzheimer’s disease-like phenotypes following chronic systemic exposure to lipopolysaccharide from *Porphyromonas gingivalis* in mice. Brain Behav. Immun..

[183] Zhang J., Yu C., Zhang X., Chen H., Dong J., Lu W., Song Z. (2018). *Porphyromonas gingivalis* lipopolysaccharide induces cognitive dysfunction, mediated by neuronal inflammation via activation of the TLR4 signaling pathway in C57BL/6 mice. J. Neuroinflamm..

[184] Cheng X., Chi L., Lin T., Liang F., Pei Z., Sun J., Teng W. (2023). Exogenous monocyte myeloid-derived suppressor cells ameliorate immune imbalance, neuroinflammation and cognitive impairment in 5xFAD mice infected with *Porphyromonas gingivalis*. J. Neuroinflamm..

[185] Ishida N., Ishihara Y., Ishida K., Tada H., Funaki-Kato Y., Hagiwara M., Ferdous T., Abdullah M., Mitani A., Michikawa M. (2017). Periodontitis induced by bacterial infection exacerbates features of Alzheimer’s disease in transgenic mice. NPJ Aging Mech. Dis..

[186] Gu Y., Wu Z., Zeng F., Jiang M., Teeling J.L., Ni J., Takahashi I. (2020). Systemic exposure to lipopolysaccharide from *Porphyromonas gingivalis* induces bone loss-correlated Alzheimer’s disease-like pathologies in middle-aged mice. J. Alzheimer’s Dis..

[187] Lei S., Li J., Yu J., Li F., Pan Y., Chen X., Ma C., Zhao W., Tang X. (2023). *Porphyromonas gingivalis* bacteremia increases the permeability of the blood-brain barrier via the Mfsd2a/Caveolin-1 mediated transcytosis pathway. Int. J. Oral Sci..

[188] Pritchard A.B., Fabian Z., Lawrence C.L., Morton G., Crean S., Alder J.E. (2022). An investigation into the effects of outer membrane vesicles and lipopolysaccharide of *Porphyromonas gingivalis* on blood-brain barrier integrity, permeability, and disruption of scaffolding proteins in a human in vitro model. J. Alzheimer’s Dis..

[189] Nonaka S., Kadowaki T., Nakanishi H. (2022). Secreted gingipains from *Porphyromonas gingivalis* increase permeability in human cerebral microvascular endothelial cells through intracellular degradation of tight junction proteins. Neurochem. Int..

[190] Qian X., Zhang S., Duan L., Yang F., Zhang K., Yan F., Ge S. (2021). Periodontitis deteriorates cognitive function and impairs neurons and glia in a mouse model of Alzheimer’s disease. J. Alzheimer’s Dis..

[191] Ilievski V., Zuchowska P.K., Green S.J., Toth P.T., Ragozzino M.E., Le K., Aljewari H.W., O’Brien-Simpson N.M., Reynolds E.C., Watanabe K. (2018). Chronic oral application of a periodontal pathogen results in brain inflammation, neurodegeneration and amyloid beta production in wild type mice. PLoS ONE.

[192] Hu Y., Li H., Zhang J., Zhang X., Xia X., Qiu C., Liao Y., Chen H., Song Z., Zhou W. (2020). Periodontitis induced by *P. gingivalis*-LPS is associated with neuroinflammation and learning and memory impairment in Sprague-Dawley rats. Front. Neurosci..

[193] Díaz-Zúñiga J., More J., Melgar-Rodríguez S., Jiménez-Unión M., Villalobos-Orchard F., Muñoz-Manríquez C., Monasterio G., Valdés J.L., Vernal R., Paula-Lima A. (2020). Alzheimer’s disease-like pathology triggered by *Porphyromonas gingivalis* in wild type rats is serotype dependent. Front. Immunol..

[194] Bahar B., Kanagasingam S., Tambuwala M.M., Aljabali A.A.A., Dillon S.A., Doaei S., Welbury R., Chukkapalli S.S., Singhrao S.K. (2021). *Porphyromonas gingivalis* (W83) infection induces Alzheimer’s disease-like pathophysiology in obese and diabetic mice. J. Alzheimer’s Dis..

[195] Tang Z., Liang D., Cheng M., Su X., Liu R., Zhang Y., Wu H. (2021). Effects of *Porphyromonas gingivalis* and its underlying mechanisms on Alzheimer-like tau hyperphosphorylation in Sprague-Dawley rats. J. Mol. Neurosci..

[196] Zhao C., Kuraji R., Ye C., Gao L., Radaic A., Kamarajan P., Taketani Y., Kapila Y.L. (2023). Nisin a probiotic bacteriocin mitigates brain microbiome dysbiosis and Alzheimer’s disease-like neuroinflammation triggered by periodontal disease. J. Neuroinflamm..

[197] Liu J., Wang Y., Guo J., Sun J., Sun Q. (2020). Salvianolic acid B improves cognitive impairment by inhibiting neuroinflammation and decreasing Aβ level in *Porphyromonas gingivalis*-infected mice. Aging (Albany NY).

[198] Nie R., Wu Z., Ni J., Zeng F., Yu W., Zhang Y., Kadowaki T., Kashiwazaki H., Teeling J.L., Zhou Y. (2019). *Porphyromonas gingivalis* infection induces amyloid-β accumulation in monocytes/macrophages. J. Alzheimer’s Dis..

[199] Leira Y., Iglesias-Rey R., Gómez-Lado N., Aguiar P., Campos F., D’Aiuto F., Castillo J., Blanco J., Sobrino T. (2019). *Porphyromonas gingivalis* lipopolysaccharide-induced periodontitis and serum amyloid-beta peptides. Arch. Oral Biol..

[200] Zeng F., Liu Y., Huang W., Qing H., Kadowaki T., Kashiwazaki H., Ni J., Wu Z. (2021). Receptor for advanced glycation end products up-regulation in cerebral endothelial cells mediates cerebrovascular-related amyloid β accumulation after *Porphyromonas gingivalis* infection. J. Neurochem..

[201] Olsen I., Singhrao S.K. (2020). *Porphyromonas gingivalis* infection may contribute to systemic and intracerebral amyloid-beta: Implications for Alzheimer’s disease onset. Expert Rev. Anti-Infect. Ther..

[202] Xue L., Zou X., Yang X.-Q., Peng F., Yu D.-K., Du J.-R. (2020). Chronic periodontitis induces microbiota-gut-brain axis disorders and cognitive impairment in mice. Exp. Neurol..

[203] Shoemark D.K., Allen S.J. (2015). The microbiome and disease: Reviewing the links between the oral microbiome, aging, and Alzheimer’s disease. J. Alzheimer’s Dis..

[204] Fulop T., Tripathi S., Rodrigues S., Desroches M., Bunt T., Eiser A., Bernier F., Beauregard P.B., Barron A.E., Khalil A. (2021). Targeting impaired antimicrobial immunity in the brain for the treatment of Alzheimer’s disease. Neuropsychiatr. Dis. Treat..

[205] French P.W. (2022). Unfolded p53 in non-neuronal cells supports bacterial etiology of Alzheimer’s disease. Neural Regen. Res..

[206] Sadrameli M., Bathini P., Alberi L. (2020). Linking mechanisms of periodontitis to Alzheimer’s disease. Curr. Opin. Neurol..

[207] Gomes A. (2021). Bacteriological Etiology of Alzheimer’s Disease: Possibility of a Vaccine?. Master’s Thesis.

[208] Jong R.A., van der Reijden W.A. (2014). Feasibility and therapeutic strategies of vaccines against *Porphyromonas gingivalis*. Expert Rev. Vaccines.

[209] O’Brien-Simpson N.M., Holden J.A., Lenzo J.C., Tan Y., Brammar G.C., Walsh K.A., Singleton W., Orth R.K.H., Slakeski N., Cross K.J. (2016). A therapeutic *Porphyromonas gingivalis* gingipain vaccine induces neutralising IgG1 antibodies that protect against experimental periodontitis. npj Vaccines.

[210] Gaspar D.P., Peres C., Florindo H.F., Almeida A.J., Ravi Kumar M.N.V. (2016). Mucosal immunization using polyester-based particulate systems. Handbook of Polyester Drug Delivery Systems.

[211] Bhardwaj P., Bhatia E., Sharma S., Ahamad N., Banerjee R. (2020). Advancements in prophylactic and therapeutic nanovaccines. Acta Biomater..

[212] Jin Z., Gao S., Cui X., Sun D., Zhao K. (2019). Adjuvants and delivery systems based on polymeric nanoparticles for mucosal vaccines. Int. J. Pharm..

[213] Azevedo A., Galhardas J., Cunha A., Cruz P., Gonçalves L.M.D., Almeida A.J. (2006). Microencapsulation of *Streptococcus equi* antigens in biodegradable microspheres and preliminary immunisation studies. Eur. J. Pharm. Biopharm..

[214] Florindo H.F., Pandit S., Gonçalves L.M.D., Videira M., Alpar H.O., Almeida A.J. (2009). Antibody and cytokine-associated immune responses to *S. equi* antigens entrapped in PLA nanospheres. Biomaterials.

[215] Florindo H.F., Pandit S., Gonçalves L.M.D., Alpar H.O., Almeida A.J. (2008). *Streptococcus equi* antigens adsorbed onto surface modified poly-ɛ-caprolactone microspheres induce humoral and cellular specific immune responses. Vaccine.

[216] Florindo H.F., Pandit S., Gonçalves L.M.D., Alpar H.O., Almeida A.J. (2009). New approach on the development of a mucosal vaccine against strangles: Systemic and mucosal immune responses in a mouse model. Vaccine.

[217] Caetano L.A., Figueiredo L., Almeida A.J., Gonçalves L.M.D. (2017). BCG-loaded chitosan microparticles: Interaction with macrophages and preliminary in vivo studies. J. Microencapsul..

[218] Wang S., Yan T., Zhang B., Chen Y., Li Z. (2024). *Porphyromonas gingivalis* vaccine: Antigens and mucosal adjuvants. Vaccines.

[219] Ferreira da Silva A., Gonçalves L.M.D., Fernandes A., Almeida A.J. (2024). Optimization and evaluation of a chitosan-coated PLGA nanocarrier for mucosal delivery of *Porphyromonas gingivalis* antigens. Eur. J. Pharm. Sci..

